# Exciton thermal radiation from macroscale membranes composed of chirality-sorted carbon nanotubes and its control

**DOI:** 10.1038/s41467-026-75711-4

**Published:** 2026-07-29

**Authors:** Akiteru Takahashi, Mioko Hizukuri, Kaichi Teranishi, Shonosuke Takaichi, Taishi Nishihara, Yuhei Miyauchi

**Affiliations:** 1 Institute of Advanced Energy, Kyoto University, Kyoto, 611-0011 Japan; 2 Department of Mechanical Engineering, The University of Tokyo, 7-3-1 Hongo, Bunkyo-ku, Tokyo, 113-8656 Japan; 3 Department of Physics, Tokyo University of Science, Kagurazaka 1-3, Shinjuku, Tokyo, 162-8601 Japan; 4 Research Institute for Science and Technology, Tokyo University of Science, Shinjuku, Tokyo, 162-8601 Japan

**Keywords:** Nanowires, Carbon nanotubes and fullerenes, Devices for energy harvesting

## Abstract

Exciton thermal radiation, which can potentially be exploited for selective thermal emission and energy harvesting, has been observed in individual single-walled carbon nanotubes (SWCNTs) heated under photoirradiation. However, whether macroscale-SWCNT assemblies can emit exciton thermal radiation under thermal conduction heating remains unclear and constitutes an important challenge for practical applications. Herein, we observe peaked exciton thermal radiation from chirality-sorted SWCNT membranes. Transmission spectroscopy shows robust exciton resonance at high temperatures, resulting in clear exciton resonance in the thermal radiation band. The absolute emissivity spectra of the membranes are determined at 850 K. Exciton dominance suppresses the contribution of thermal free carriers to the infrared absorption/emission spectra, maintaining the transparency below the optical gap even at elevated temperatures. Furthermore, we demonstrate the enhancement of emissivity at the exciton resonance using a simple planar few-layer architecture consisting of alternating SWCNT and transparent dielectric layers, enabled by strong excitonic light-matter interactions in SWCNTs; this offers a pathway toward superior spectral selectivity at even higher temperatures. These results highlight the potential of chirality-sorted SWCNT membranes as a class of semiconductors for controlling thermal radiation at elevated temperatures, leveraging thermo-optical properties that differ from those of conventional bulk semiconductors.

## Abstract (web summary)

Exciton thermal radiation could improve energy harvesting, but its observation in macroscale assemblies remains challenging. Here, the authors demonstrate peaked exciton thermal radiation from carbon nanotube membranes under thermal conduction.

## Introduction

**Fig. 1:**
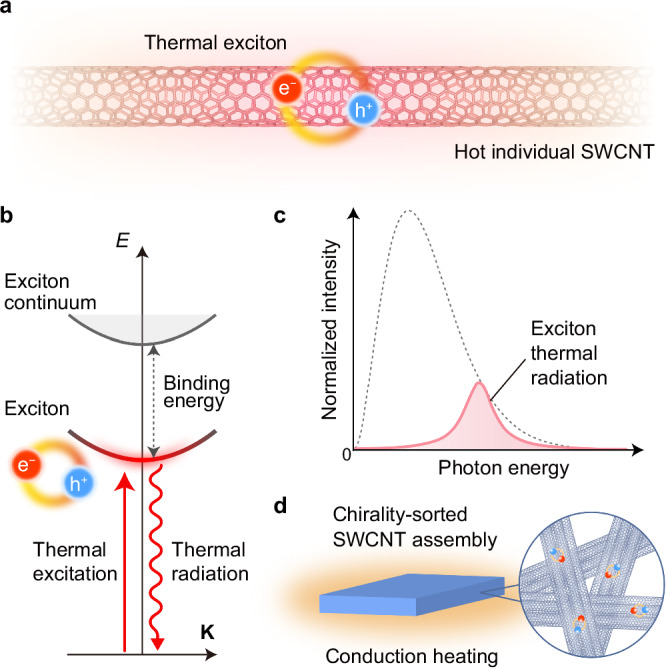
Schematics of thermal exciton in carbon nanotubes. **a** A thermal exciton (a bound electron–hole pair) in a hot individual single-walled carbon nanotube (SWCNT). **b** Exciton and continuum energy levels in SWCNTs (E, energy; ${{\bf{K}}}$, wavenumber vector). **c** Spectral features of exciton thermal radiation from an individual SWCNT (red curve) and blackbody radiation (dotted curve). The peak intensity of exciton thermal radiation is normalized to the blackbody radiation curve. **d** Excitons in a membrane of assembled chirality-sorted SWCNTs.

Excitons are hydrogen-atom-like quasiparticles in which an electron and a hole are bound through an attractive Coulomb interaction. As the elementary excitations in semiconductors, excitons have long been a focal topic in photophysics research. The exciton signature is a sharp peak below the band edge in optical spectra^1^. The binding energy of excitons, the energy required for dissociating excitons into free electrons and holes, is typically of the order of a few tens of meV^1^ in conventional bulk semiconductors. Consequently, distinct exciton features in the optical spectra of bulk semiconductors are difficult to observe at room temperatures (≈ 26 meV) or higher, limiting the study and application of excitons at elevated temperatures. This historical paradigm has been shifted by the emergence of nanomaterials such as single-walled carbon nanotubes (SWCNTs; Fig. 1a)^2^ and transition-metal dichalcogenides^3^. Within these inherently low-dimensional materials, the carriers are spatially confined and Coulomb screening is weak, boosting the exciton binding energies to several hundred meV^4–8^ and enabling the generation of thermal excitons with observable thermal radiation^9–12^ (Fig. 1b). In contrast to the typical blackbody or graybody thermal radiation from bulk semiconductors, exciton thermal radiation exhibits a narrow linewidth^9,13^ (Fig. 1c) originating from the atom-like well-defined energy levels of the excitons. Accordingly, the recombination of thermally excited excitons is followed by the emission of photons with an energy corresponding to the exciton energy (Fig. 1b). The exciton thermal radiation of SWCNTs in the near-infrared range has aroused particular interest because it can potentially enhance the energy efficiency of applications such as wavelength-selective emitters for thermophotovoltaic (TPV) power generation^14,15^. To effectively generate electricity, a photovoltaic cell requires thermal radiation with a controlled bandwidth from the hot emitter.

SWCNTs used as thermal light emitters in real applications must be assembled into macroscale materials and heated through conduction (Fig. 1d). To date, advances in dispersion and separation methods for each (*n*, *m*) structure (chirality) of SWCNTs^16–20^ have enabled the production of high-purity chirality-sorted SWCNT assemblies as emerging opto-functional materials^21–31^. However, in all previous studies, the peaked light emission spectra have been measured from individual SWCNTs heated under photoirradiation or electronic current injection, which is inevitably accompanied by nonthermal excitation^9,10,32,33^. Whether purely thermal exciton radiation can be observed from an SWCNT assemblage via conductive heat transfer remains an open question. Experimentally, this has remained challenging due to technical difficulties in isolating purely thermal radiation from thin membranes under thermal conduction heating from thermal radiation of any other radiation sources, such as heaters. Furthermore, just because exciton thermal radiation can be observed for individual SWCNTs does not necessarily mean that it can be observed in macroscale SWCNT membranes. Various factors can potentially prevent observations of exciton thermal radiation from bulk assemblies. Although individual SWCNTs exhibit sharp exciton resonances, the absorptivity contrast between resonant and nonresonant photon energies in macroscale SWCNT membranes can diminish with increasing membrane thickness, obscuring the exciton resonance in thermal radiation spectra. Moreover, the exciton binding energy can be lower in assembled SWCNTs than in individual SWCNTs because the Coulomb screening in the assemblage is enhanced by the dielectric responses of the surrounding SWCNTs (Fig. 1d). At high temperatures, the exciton resonance peak is additionally broadened by enhanced exciton phonon scattering^9,10,34^. These factors can attenuate the exciton resonance in the optical spectra of assembled SWCNTs at high temperatures. Meanwhile, as the exciton resonance energy depends on chirality, SWCNT assemblies should comprise the same-chirality SWCNTs^35,36^. A mixed-chirality SWCNT assembly exhibits a relatively featureless optical spectrum, including thermal radiation^37–43^. Therefore, determining whether exciton thermal radiation can be observed from the assemblies of chirality-sorted SWCNTs under thermal conduction heating and elucidating the high-temperature optical properties of these materials are essential for fundamental science and technological applications.

Here, we report the observations of exciton thermal radiation from chirality-sorted SWCNT membranes. To this end, we prepared a dispersion enriched with SWCNTs with chiral indices of (7,5)^16^ and fabricated a membrane from the dispersion via vacuum filtration methods^30^. First, we transferred the SWCNT membrane onto a sapphire substrate, heated it with a ceramic heater, and obtained its transmittance spectra. After the initial heating and cooling process, the exciton peak showed a reversible spectral change in the temperature range 300–800 K. As the temperature increased, the exciton peak exhibited red-shifting and linewidth broadening while the integrated oscillator strength remained constant, indicating high-temperature robustness of the exciton state in the SWCNT membrane. The peaked thermal radiation spectra were observed during heating of the free-standing membrane with a tungsten heater specifically designed for this study. At 850 K, the spectral shapes of the emissivity peaks agreed with the predicted absorptivity, implying that the peak of the thermal radiation was contributed by excitons. The robustness of the excitons, even at 850 K, suppressed the infrared absorptivity and emissivity arising from free carriers. This situation contrasts with that in bulk semiconductors^44,45^. Our thin chirality-sorted SWCNT membranes present as a material showing the optical characteristics of nearly ideal two-dimensional sheets even at high temperatures; meanwhile, the one-dimensional nature of the electronic states in each individual SWCNT ensures the dominance of exciton resonance. Furthermore, these characteristics enable the enhancement of exciton thermal radiation through a simple planar four-layer architecture consisting of alternating SWCNT and transparent dielectric layers. This design offers a pathway toward superior spectral selectivity at even higher temperatures, as demanded for efficient TPV power generation and other high-temperature photonics applications that exploit exciton resonances with high thermal stability.

## Results

### Macroscale membranes of SWCNTs

**Fig. 2:**
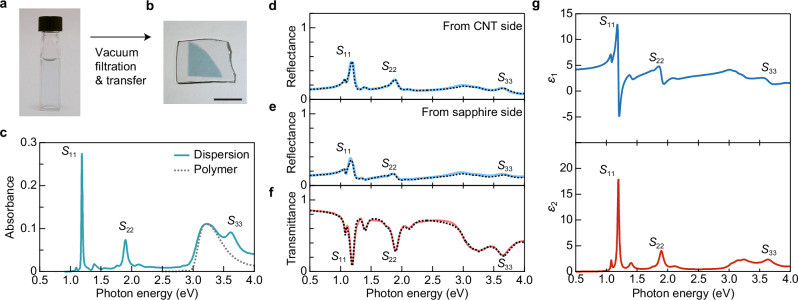
Characterization of the chirality-sorted carbon nanotube samples. The (7,5) carbon nanotube (CNT) dispersion (**a**) and the membrane transferred onto a sapphire substrate (**b**) (scale bar = 5 mm). **c** Absorption spectrum of the (7,5) CNT dispersion in an optical cell with a path length of 10 mm (green solid curve). For reference, the absorption spectrum of the dispersant polymer at 0.24 µg mL^−1^ (gray dotted curve) is also plotted. *S*_*ii*_ is the *i*th subband of the exciton state of (7,5) CNTs. **d–f** Reflectance (**d, e**: blue curves) and transmittance (**f**: red curve) spectra of the (7,5) CNT membrane. The reflectance spectra are obtained in two configurations: incident light entering from the CNT membrane side (**d**) and incident light entering from the sapphire substrate (**e**). The dotted black curves are the fitting results. **g** Complex dielectric function ($\tilde{\varepsilon }={\varepsilon }_{1}+{{\rm{i}}}{\varepsilon }_{2}$) of the (7,5) CNT membrane (${\varepsilon }_{1}$: blue, ${\varepsilon }_{2}$: red curves, respectively). Source data are provided as a Source Data file.

The single-chirality SWCNT membranes were fabricated and characterized as follows (Methods). Briefly, a blue-colored dispersion enriched with (7,5) SWCNTs (Fig. 2a) was separated from the mixed samples using the organic polymer poly(9,9-dioctylfluorenyl-2,7-diyl) (PFO)^16^ as a dispersant. The dispersion was vacuum filtered to fabricate the membrane, which was then transferred onto a sapphire substrate (hereafter referred to as an on-sapphire membrane) for optical characterizations and thickness measurements (Fig. 2b). The absorption spectrum of the dispersion (Fig. 2c) displays the clear peaks of the first (*S*_11_), second (*S*_22_) and third (*S*_33_) subband exciton states of the (7,5) SWCNTs at 1.186, 1.898 and 3.614 eV, respectively^35^. The minor peak at 1.092 eV arises from the *S*_11_ exciton state of (7,6) SWCNT^35^, which is minorly contained in the dispersion. Following ref. ^46^, the concentrations of (7,5) and (7,6) SWCNTs were determined as 0.66 and 0.02 µg mL^−1^, respectively, clarifying that the dispersion was composed of almost pure (7,5) SWCNTs and the dispersant polymer molecules. The broad peak at 3.220 eV is attributed to absorption of the dispersant polymer. For reference, the absorption spectrum of the dispersant polymer (0.24 µg mL^−1^) is also displayed in Fig. 2c. As their absorption peaks did not decrease after repeated procedures to remove the free polymers (Methods), the residual polymers were assigned to those wrapped around SWCNTs.

Figure 2d–f shows the reflectance and transmittance spectra of the on-sapphire SWCNT membranes. The reflectance spectra were obtained in two configurations, one with the incident light entering from the SWCNT membrane side (Fig. 2d) and the other with light entering from the sapphire substrate (Fig. 2e). The reflection spectra are distinct because the refractive index at the air–SWCNT interface differs from that at the sapphire–SWCNT membrane interface. All optical spectra display the signatures of the *S*_11_ and *S*_22_ exciton states of (7,5) SWCNTs at photon energies slightly below (a few meV) those in the spectrum of the dispersion. To determine the complex dielectric function (Fig. 2g) and membrane thickness (53 nm), we fitted all spectra to the model functions of the individual features in the optical spectra of SWCNTs^30^ (Methods). The dispersion spectrum dominantly featured the *S*_11_ exciton resonance of (7,5) SWCNTs; the peak structures of other chirality SWCNTs were absent except for a small peak attributed to residual (7,6) SWCNTs at a photon energy below that of the *S*_11_ exciton states. This observation further supports the high purity of the (7,5) SWCNTs.

### Temperature dependence of the transmittance spectra

**Fig. 3:**
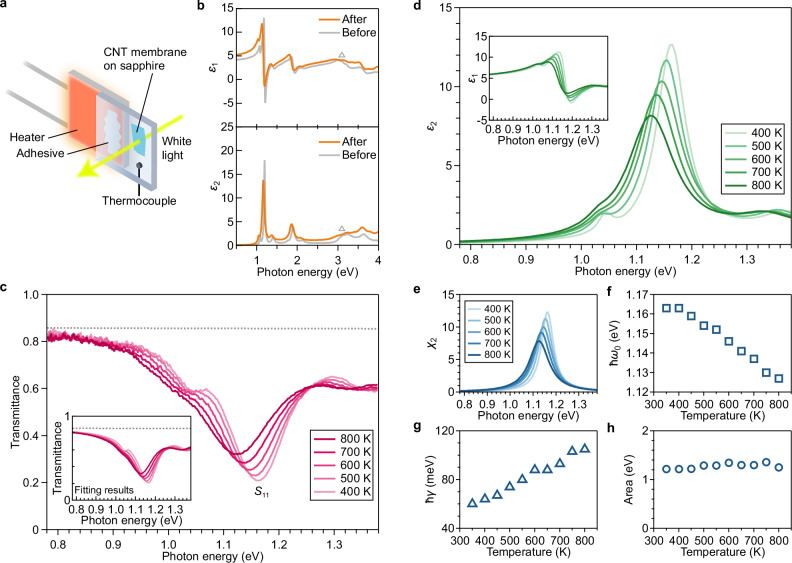
Temperature dependence of transmittance. **a** Schematic of the optical setup for high-temperature transmittance measurements of a carbon nanotube (CNT) membrane. **b** Complex dielectric function ($\tilde{\varepsilon }={\varepsilon }_{1}+{{\rm{i}}}{\varepsilon }_{2}$) of the (7,5) CNT membrane before (gray) and after (orange) three heating–cooling cycles. Open triangles indicate the absorption peak position of the polymers. **c** Temperature dependence of the transmittance spectra around the first subband (*S*_11_) exciton and the sapphire substrate (gray dotted lines), along with the fitting results (inset). Temperature dependences of **d** ${\varepsilon }_{1}$ (inset) and ${\varepsilon }_{2}$ obtained from the fittings and **e** the imaginary part of the optical susceptibility ($\tilde{\chi }={\chi }_{1}+{{\rm{i}}}{\chi }_{2}$) of the *S*_11_ exciton of (7,5) CNTs, which can be described by a Lorentzian function $\tilde{\chi }\left(\omega \right)=f{\left[\left({\omega }_{0}^{2}-{\omega }^{2}\right)-{{\rm{i}}}\omega \gamma \right]}^{-1}$ with f, ${\omega }_{0}$, and γ being the strength, resonant optical frequency, and damping term, respectively. In **c**–**e**, the color of the spectra grades from light to dark as the temperature increases. Temperature dependence of **f** ${\hbar \omega }_{0}\,$(ħ = reduced Planck constant), **g** $\hbar \gamma,$ and **h** area. Source data are provided as a Source Data file.

To determine the fundamental optical properties of the (7,5) SWCNT membrane at high temperatures, we examined the high-temperature exciton response via transmittance spectroscopy. For this purpose, the SWCNT membrane was placed on one side of the sapphire substrate and the opposite side of the substrate was glued to the ceramic heater (Fig. 3a). The sample temperatures were measured by a thermocouple positioned in the immediate vicinity of the SWCNT membrane. The SWCNT membrane was irradiated with collimated white light from a halogen lamp and its transmittance was measured between 300 and 800 K throughout three cycles of heating and cooling (Methods). The transmittance spectrum was irreversibly altered during the initial heating–cooling cycle but reversibly altered during the second and third cycles (Supplementary Fig. 1). Figure 3b shows the complex dielectric functions of the SWCNT membrane at 300 K before (gray curves; also plotted in Fig. 2g) and after the heating–cooling processes (orange curves) (here, the complex dielectric functions were derived from the optical spectra shown in Supplementary Fig. 2). The exciton peaks show redshifts and broadened linewidths, and the absorption peaks of the polymers (open triangles) are diminished. Moreover, the membrane thickness reduced from 53 to 38 nm. These results indicate decomposition of the polymers and a consequent reduction in thickness. The redshift of the *S*_11_ exciton peak indicates that as the polymer molecules decomposed, close contact among the nanotubes altered the surrounding dielectric environment.

Figure 3c shows the transmittance spectra of the on-sapphire SWCNT membrane during the third temperature cycle at different temperatures. As the temperature increased, the exciton peaks became more redshifted, their intensities reduced, and their linewidths broadened. From the transmittance spectrum at different temperatures, we obtained the complex dielectric functions ($\tilde{\varepsilon }={\varepsilon }_{1}+{{\rm{i}}}{\varepsilon }_{2}$) at each temperature. More specifically, the transmittance spectra were fitted to a model complex dielectric function obtained by incrementally modifying the parameters describing the *S*_11_ exciton and its phonon sideband, which were initially set at room temperature. The simulation results (Fig. 3c, inset) well reproduce the experimental results (Supplementary Fig. 3). Figure 3d shows the temperature dependence of the imaginary part of the dielectric function (${\varepsilon }_{2}$) and its corresponding real part (${\varepsilon }_{1}$; inset). Between 400 and 800 K, the dominant exciton peak exhibits an approximately 60% decrease in intensity and an approximately twofold increase in full-width-at-half-maximum. Linewidth broadening weakened the photon-energy dependence of the real part (inset). Figure 3e plots the temperature dependence of the optical susceptibility ($\tilde{\chi }={\chi }_{1}+{{\rm{i}}}{\chi }_{2}$) of the *S*_11_ exciton of the (7,5) SWCNTs contributing to the imaginary part of the complex dielectric function. The spectral shape is well reproduced by a susceptibility function based on the Lorentz model, given by $\tilde{\chi }\left(\omega \right)=f{\left[\left({\omega }_{0}^{2}-{\omega }^{2}\right)-{{\rm{i}}}\omega \gamma \right]}^{-1}$, where f, ${\omega }_{0}$, and γ are the strength, exciton resonant optical frequency, and damping term, respectively. Figure 3f–h summarizes the ${\hbar \omega }_{0}$, $\hbar \gamma$, and area of the imaginary part ${\chi }_{2}$ obtained from fitting, respectively (${{\rm{where}}}\,\hbar$ is the reduced Planck constant). The $\hbar {\omega }_{0}$ and $\hbar \gamma$ parameters are temperature-dependent (as observed above) but the area, which corresponds to the oscillator strength, is almost independent of temperature (Fig. 3h). Therefore, even under the aggregation conditions of the membrane, the excitons in the SWCNTs are thermally stable against dissociation into free carriers at 800 K and retain their oscillator strength. This thermal stability is also supported by the recently reported robust exciton binding energy in SWCNT membranes^47^.

### Observation of exciton thermal radiation

**Fig. 4:**
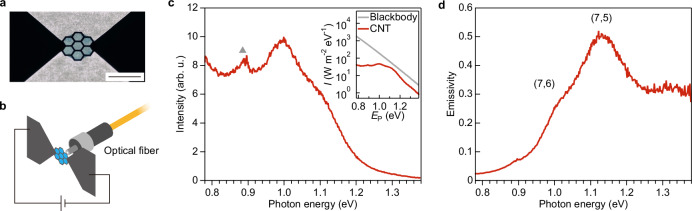
Thermal radiation of the carbon nanotube membrane. **a** Photograph of the carbon nanotube (CNT) membrane on the specially designed tungsten plate (scale bar = 5 mm). **b** Schematic of the optical setup for measuring thermal radiation. **c** Thermal radiation spectrum and **d** absolute spectral emissivity of the CNT membrane. In **c**, the gray triangle indicates the artifact from the optical fiber and the inset shows the spectra of the CNT membrane (red curve) and a blackbody (gray curve) at 850 K (log–linear scale). *I* and *E*_P_ denote the spectral emissive power and photon energy, respectively. Source data are provided as a Source Data file.

Because exciton resonance in the complex dielectric function is retained at high temperatures, the SWCNT membrane is expected to emit exciton thermal radiation. For this observation, we prepared a free-standing SWCNT membrane on a specially designed heater, namely, a thin (0.1 mm) tungsten plate (Fig. 4a). Figure 4b is a schematic of the optical setup for observing radiation from the SWCNT membrane. When a voltage is applied across the tungsten plate, Joule heat is mainly generated around the honeycomb-structured part. The resulting thermal energy is then conducted to the SWCNT membrane (Methods). Numerical simulations confirmed that the temperature of the SWCNT membrane suspended over the central hexagonal part of the heater is nearly uniform throughout the membrane, as shown in Supplementary Fig. 4 (Methods). The thermal radiation of the SWCNT membrane was detected through an optical fiber placed near the membrane to avoid detection of radiation from any other thermal radiation sources. The temperature of the SWCNT membrane was determined from the spectral shape of the emissivity spectrum obtained from the thermal radiation spectrum (Methods). Briefly, using the complex dielectric functions obtained from the experiments above (Fig. 3d), we calculated the emissivity spectra in the 350–800 K range (Supplementary Fig. 5). The resulting emissivity spectra revealed that the intensity ratio of the exciton peak and its phonon side peaks is almost constant. The temperature at which the emissivity spectrum obtained from the measured thermal radiation spectrum divided by the Planck function satisfied the intensity-ratio characteristic was determined as the temperature of the SWCNT membrane. Figure 4c, d show the thermal radiation spectrum and the absolute spectral emissivity of the free-standing SWCNT membrane, respectively, at ≈ 850 K. The thermal radiation spectrum exhibits a peak at 1.0 eV and a shoulder peak at around 1.1–1.2 eV. The emissivity spectrum similarly shows a major peak structure at 1.1–1.2 eV arising from the *S*_11_ exciton states of the (7,5) SWCNTs, together with a small peak around 1.0 eV produced by residual (7,6) SWCNTs. From these results, the thermal radiation peaks at 1.1–1.2 and 1.0 eV in the thermal radiation spectrum (Fig. 4c) were attributed to exciton resonances of the (7,5) and (7,6) SWCNTs, respectively. Even under thermal conduction heating, the excitons in the SWCNT membrane were thermally excited and recombined, and thereafter emitted photons at an energy corresponding to the exciton energy. At ≈ 850 K, the intensity of the thermal radiation peak assigned to the *S*_11_ exciton resonance from (7,6) SWCNTs exceeded that from (7,5) SWCNTs (Fig. 4c). This is because (7,6) SWCNTs, having a lower exciton energy, are more easily thermally excited than (7,5) SWCNTs.

**Fig. 5:**
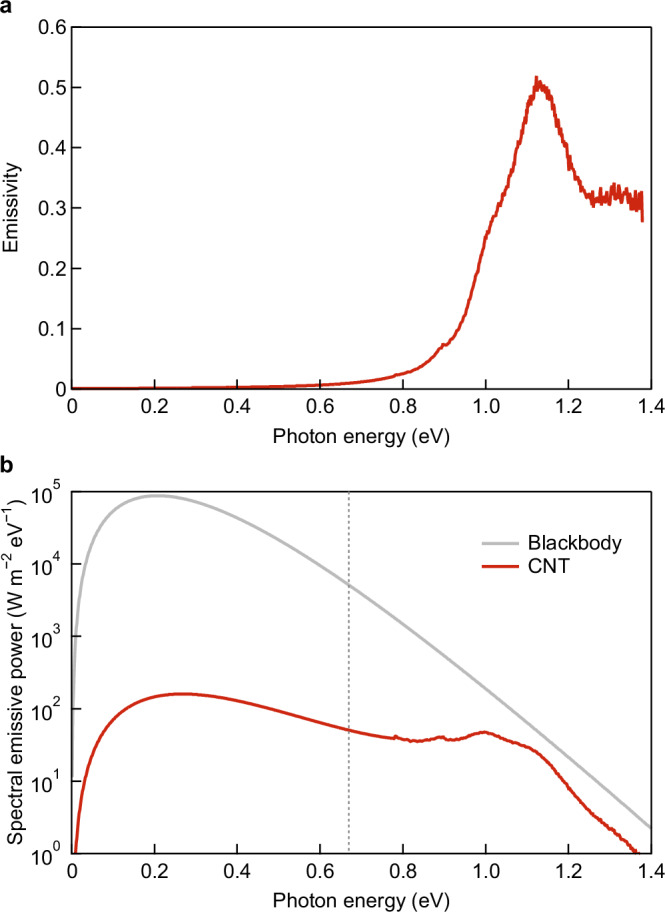
Broadband optical spectra. **a** The absolute spectral emissivity of the free-standing (7,5) carbon nanotube (CNT) membrane at 850 K. **b** Calculated thermal radiation spectra of the CNT membrane (red curve) and a blackbody (gray curve) at 850 K (logarithmic–linear plot). The dotted line indicates a bandgap energy of a typical GaSb photovoltaic cell of 0.67 eV. Source data are provided as a Source Data file.

The emissivity was strongly suppressed at photon energies below the *S*_11_ exciton states of (7,5) and (7,6) SWCNTs, even at ≈ 850 K (Fig. 4d). In contrast, the emissivity of conventional semiconductors is typically increased below the optical gap because free carriers are absorbed and emitted at high temperatures (e.g., the optical gap of silicon totally disappears at ≈ 800 K^44,45^). Consequently, the radiation intensities at photon energies below the exciton states are largely suppressed from that of blackbody radiation at the same temperature (Fig. 4c, inset). Figure 5a shows the absolute spectral emissivity of the SWCNT membrane at ≈ 850 K extrapolated to the infrared region (Methods), and Fig. 5b compares the thermal radiation spectra of the SWCNT membrane and a blackbody at 850 K on a log–linear scale. The membrane radiation is much less intense than the blackbody radiation at the lower-energy side, especially at the blackbody radiation peak at 200 meV, indicating the higher spectral selectivity of near-infrared thermal radiation from the SWCNT membrane. The proportion of integrated radiation energy above 0.67 eV (the estimated bandgap energy of the GaSb photovoltaic cell typically used in TPV energy conversion) which is indicated as a dotted line in Fig. 5b, is ≈ 20% of the total radiated energy in the 0.05–1.36 eV range of photon energies. This spectral selectivity^48^ is an order of magnitude higher than blackbody’s spectral selectivity (≈ 2%) at the same temperature. This high value as an intrinsic material is obtained without controlling the radiation field through photonic fine structures, highlighting the attractiveness of chirality-sorted SWCNT membranes as wavelength-selective emitter materials in the near-infrared region. Additionally, as shown in Fig. 5a, the emissivity of the exciton resonance peak is 0.5, which coincides with the maximum emissivity and absorptivity of an ultimately two-dimensional sheet with a high absorption coefficient^49^. This finding suggests that, whereas a one-dimensional electronic system of SWCNTs ensures the thermal stability of excitons, thin SWCNT membranes behave as nearly ideal two-dimensional sheets with strong absorption of near-infrared light.

### Enhanced excitonic emissivity in few-layer architectures

**Fig. 6:**
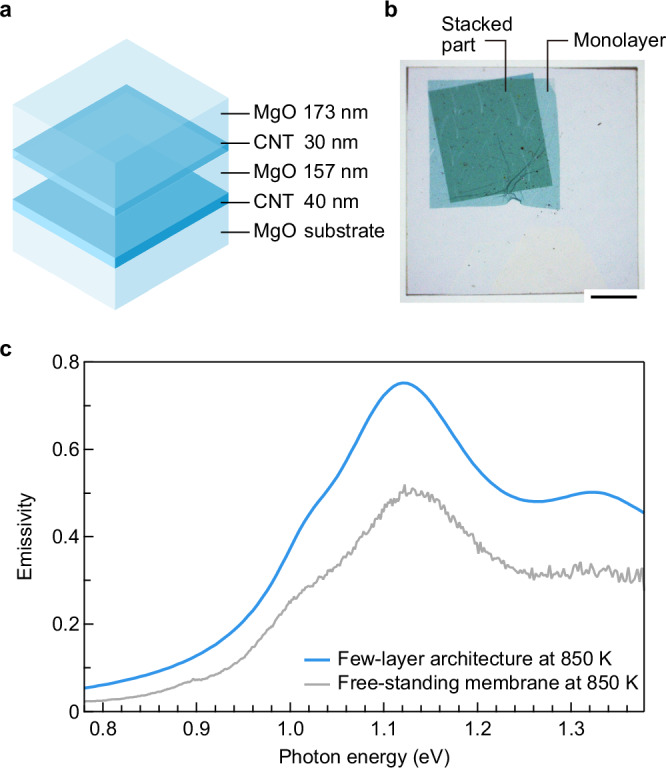
Carbon nanotube few-layer architecture. **a, b** A schematic (**a**) and a photograph (**b**) of the (7,5) carbon nanotube (CNT)–MgO few-layer architecture (scale bar = 2 mm). **c** The emissivity spectra of the fabricated few-layer architecture (blue) and the free-standing membrane (gray) at 850 K. The spectrum of the few-layer architecture is obtained from its transmittance spectrum at 850 K. The spectrum of the free-standing membrane is the same as the one shown in Fig. 5a. Source data are provided as a Source Data file.

Here, we demonstrate that a thin SWCNT-membrane is an optofunctional material potentially enabling an unconventional photonics strategy for controlling near-infrared thermal radiation. As discussed above, the SWCNT membrane inherently possesses a high emissivity contrast between the exciton resonance and off-resonance photon energies, yielding high spectral selectivity as an intrinsic material. However, the single-layer membrane can never exceed the theoretical emissivity limit of 0.5 for a two-dimensional film^49^ at its resonance peak. Furthermore, utilizing a standalone free-standing membrane with a thickness of less than 50 nm as a practical thermal emitter presents significant structural challenges. To overcome these fundamental and practical limitations, here we implement a planar few-layer architecture consisting of alternating SWCNT and lossless dielectric layers as shown in Fig. 6a. This architecture was recently proposed to realize near-perfect absorption at the exciton resonance of the SWCNT membranes via destructive interference between the reflected fields from the front and back SWCNT layers, separated by a transparent dielectric spacer; its feasibility was experimentally demonstrated at room temperature^50^. In this study, we extend this strategy to achieve a significant enhancement of excitonic emissivity at elevated temperatures. Specifically, we designed the few-layer structure comprising (7,5) SWCNT membranes and thermally stable, infrared-transparent MgO layers to enhance exciton emissivity at 850 K (Fig. 6a) and characterized its optical spectra at this temperature. Figure 6b shows a photograph of the fabricated few-layer architecture using two (7,5) SWCNT membranes. Compared to the monolayer part, the blue color is more intense at the stacked part. Figure 6c shows the emissivity spectrum at 850 K, which was derived via the temperature-dependent transmittance spectroscopy (Methods). The emissivity at the exciton resonance was enhanced by more than 1.5 times compared to that of a single-layer membrane. This result demonstrates the feasibility of using few-layer planar architecture to significantly enhance excitonic emissivity beyond the 0.5 limit, even at elevated temperatures.

## Discussion

**Fig. 7:**
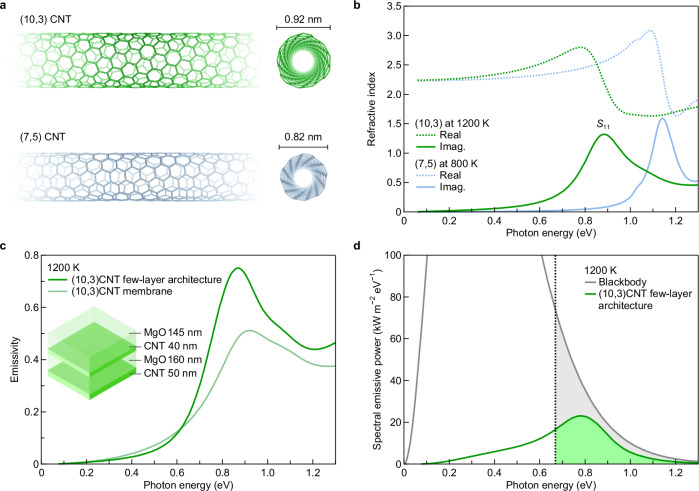
Wavelength-selective thermal emitter. **a** Structures of (10,3) and (7,5) carbon nanotubes (CNTs). The right panels show the corresponding cross-sectional views. **b** Simulated complex refractive index spectra of (10,3) CNT membrane at 1200 K (green curves), compared with the measured complex refractive index spectra of the (7,5) CNT membrane at 800 K (blue curves). The *S*_11_ is the first subband of the exciton state of (10,3) CNT. **c, d** Simulated emissivity (**c**: dark green) and thermal radiation (**d**: green) spectra of the few-layer architecture (the dimensions are shown in the inset of **c**) at 1200 K. In **c**, the light green curve shows the emissivity spectrum of a free-standing (10,3) membrane with a thickness of 90 nm. In **d**, the gray curve shows the blackbody radiation spectrum at 1200 K. The dotted line indicates a bandgap energy of a typical GaSb photovoltaic cell of 0.67 eV. Source data are provided as a Source Data file.

Finally, using our findings as a foundation, we outline possible strategies for constructing practical wavelength-selective emitters to facilitate efficient TPV energy conversion. One primary requirement is elevating the operating temperature, as 850 K remains relatively low for typical far-field TPV systems. Given that the inherent strain in small-diameter SWCNTs limits their thermal stability, utilizing larger-diameter SWCNTs is an approach to enhance structural robustness at higher temperatures. To this end, we theoretically investigate the potential of a few-layer architecture employing (10,3) SWCNTs (Fig. 7a), which have a larger diameter than (7,5) SWCNTs and remain thermally robust even above 1200 K. Figure 7b shows the complex refractive index spectrum of (10,3) SWCNTs at 1200 K predicted using the empirical formulas^30,51^ (Methods). Due to the larger diameter, the *S*_11_ exciton resonance energy, which scales roughly inversely with diameter, shifts to lower energies and is further red-shifted by the temperature-induced band gap reduction. Figure 7c shows the predicted emissivity spectrum of the few-layer architecture optimized for 1200 K (the inset shows the device dimensions). The excitonic emissivity reaches 0.74 even at this elevated temperature, while the off-resonance infrared emissivity remains even lower than that of a single-layer (10,3) membrane. Consequently, the predicted thermal radiation spectrum (Fig. 7d) reveals that the integrated power (≈ 10 kW m^−2^) is an order of magnitude higher than that of unconcentrated sunlight (1 kW m^−2^), fulfilling the requirements for efficient operation of typical PV cells (Supplementary Fig. 6). Notably, the predicted spectral selectivity is significantly enhanced to 64% (it is only 12% for a blackbody emitter), suggesting that this architecture is a viable candidate for practical wavelength-selective emitters with high spectral selectivity.

In conclusion, our results highlight fundamental distinctions between the high-temperature optical properties of chirality-sorted SWCNT membranes and conventional semiconductors. Owing to their characteristics, SWCNT membranes can sustain sharp exciton resonances even at high temperatures. Furthermore, the enhancement of excitonic emissivity using a simple few-layer architecture has been demonstrated at elevated temperatures, facilitating energy harvesting based on thermal photonics technologies relying on the excitonic properties of SWCNTs.

## Methods

### Materials

CoMoCAT SWCNTs (>95% carbon basis, >95% as carbon nanotubes, average diameter 0.78 nm; Sigma-Aldrich Co. LLC), poly(9,9-dioctylfluorenyl-2,7-diyl) (PFO; Sigma-Aldrich Co. LLC), and toluene (>99.5%; FUJIFILM Wako Pure Chemical Corp.) were used as received without further purification.

### Dispersion preparation

(7,5) SWCNTs were selectively separated from CoMoCAT SWCNTs in toluene using PFO polymer as the dispersant^16^. The PFO (40 mg) was dissolved in 40 mL toluene and then well mixed with 20 mg of the SWCNT raw material through bath sonication (60 min), followed by tip sonication (3 h) at 24 W. The mixed dispersion was ultracentrifuged at 19,000× *g* for 30 min, and the (7,5) SWCNT dispersion was obtained as the supernatant. To remove excessive free PFO molecules, the dispersion was vacuum-filtered on a hydrophilic MCE membrane filter with a pore size of 0.05 µm (JVWP02500, MERCK). The SWCNTs on the membrane filter were then re-dispersed in toluene, and their absorbance spectra were measured in an ultraviolet–visible–near-infrared (UV–Vis–NIR) spectrometer (V-770, JASCO). For UV–Vis–NIR measurements, the sample was dispensed into an optical cell with a path length of 10 mm.

### Membrane preparation

The SWCNT membranes were fabricated via vacuum filtration^30^. The dispersion was filtered under a vacuum pressure of 70–80 kPa on a hydrophilic mixed cellulose ester membrane filter with a pore size of 0.05 µm (VSWP02500, MERCK) and then dried at 3 kPa for 1 h. Subsequently, the pressure was elevated to 30 kPa, and toluene was added for washing. The membrane was again dried at 3 kPa for 1 h. The SWCNT membrane on the filter was cut and transferred to the sapphire, metal washer, and a tungsten plate via wet-transfer processes. The optical spectra of the on-sapphire membranes were measured in the abovementioned UV–Vis–NIR spectrometer.

### Complex dielectric function spectrum

The complex dielectric function spectrum of the SWCNT membrane was determined according to ref. ^30^. First, the microscopic optical susceptibility$\,\tilde{\chi }\,$ of an SWCNT membrane of bulk density ρ was modeled as follows:

1 $$\tilde{\chi }\left(\rho,\omega \right)=\rho \left[{\sum }_{i}{\tilde{\chi }}_{{{\rm{L}}}}^{i}\left(\omega \right)+{\tilde{\chi }}_{{{\rm{C}}}}\left(\omega \right)+{\tilde{\chi }}_{{{\rm{D}}}}\left(\omega \right)+{\chi }_{{{\rm{B}}}}\right]$$

where ω is the optical frequency.

The first term on the right-hand side of Eq. (1), ${\tilde{\chi }}_{{{\rm{L}}}}^{i}$, is a Lorentzian function of the *i*th peaked oscillator (where the oscillators correspond to excitons and phonon sidebands). It is calculated as follows:

2 $${\tilde{\chi }}_{{{\rm{L}}}}^{i}\left(\omega \right)={f}_{{{\rm{L}}}}^{i}{\left[\left({{\omega }_{{{\rm{L}}}}^{i}}^{2}-{\omega }^{2}\right)-{{\rm{i}}}\omega {\gamma }_{{{\rm{L}}}}^{i}\right]}^{-1}$$

where ${f}_{{{\rm{L}}}}^{i}$, ${\omega }_{{{\rm{L}}}}^{i}$, and ${\gamma }_{{{\rm{L}}}}^{i}$ are the strength, resonant optical frequency, and damping term of the *i*th Lorentz oscillator, respectively.

The second term ${\tilde{\chi }}_{{{\rm{C}}}}$ on the right-hand side of Eq. (1), represents the nearly featureless continuum band in the photon-energy region above the *S*_11_ exciton resonance. This term is given by a phenomenological step-like function as follows:

3 $${\tilde{\chi }}_{{{\rm{C}}}}\left(\omega \right)=	{A}_{{{\rm{C}}}}\left[G\left(\left(\omega -{\omega }_{{{\rm{C}}}}\right){\gamma }_{{{\rm{C}}}}^{-1}\right)+G\left(\left(\omega+{\omega }_{{{\rm{C}}}}\right){\gamma }_{{{\rm{C}}}}^{-1}\right)\right.\\ 	 \left. -G\left(\left(\omega -{\omega }_{{{\rm{cut}}}}\right){\gamma }_{{{\rm{cut}}}}^{-1}\right)-G\left(\left(\omega+{\omega }_{{{\rm{cut}}}}\right){\gamma }_{{{\rm{cut}}}}^{-1}\right)\right]$$

where $G\left(x\right)$ is a series of π/2-phase-shifted Lorentzian functions $g\left(x\right)={\left(x+{{\rm{i}}}\theta \right)}^{-1}$; in particular, $G\left(x\right)={\sum }_{p=0}^{100}2{{\rm{i}}}g\left(x,\left(p+0.5\right){{\rm{\pi }}}\right),$ where p is the summation index; ${A}_{{{\rm{c}}}}$ is the absorption strength; and ${\omega }_{{{\rm{c}}}}$ and ${\gamma }_{{{\rm{C}}}}^{-1}$ are parameters specifying the center frequency and gradient at ${\omega=\omega }_{{{\rm{C}}}}$, respectively. The cutoff energy and gradient are given by $\hbar {\omega }_{{{\rm{cut}}}}$ = 6 eV and ${\left(\hbar {\gamma }_{{{\rm{cut}}}}\right)}^{-1}$ = 1 eV^−1^, respectively.

The third term ${\tilde{\chi }}_{{{\rm{D}}}}\,$ on the right-hand side of Eq. (1) represents the Drude-like response of free carriers. We set ${\tilde{\chi }}_{{{\rm{D}}}}\left(\omega \right)=0$ because our membrane showed no Drude-like response at lower photon energies (Fig. 2d–f). The fourth term ${\chi }_{{{\rm{B}}}}\,$ on the right-hand side of Eq. (1) is the background susceptibility (a real constant) describing all contributions other than the abovementioned oscillators. The corresponding complex relative dielectric function ($\tilde{\varepsilon }$) is given as $\tilde{\varepsilon }\left(\rho,\omega \right)=1+\tilde{\chi }(\rho,\omega )$.

The reflection and transmission spectra of the SWCNT membrane with $\tilde{\varepsilon }\left(\rho,\omega \right)$ were calculated using the optical transfer matrix technique^52^. The complex refractive index spectrum of sapphire was taken from ref. ^53^. For the on-sapphire SWCNT membrane, the back surface reflection must also be considered ^30^. Here, ρ was set to 1 g cm^−3^ (the typical density of SWCNT membranes^30^) and $\tilde{\varepsilon }\left(\rho,\omega \right)$ was obtained by determining the above oscillator parameters that reproduce all reflectance and transmittance spectra of the SWCNT membrane (Fig. 2d–f).

### Temperature-variable transmittance spectroscopy

The SWCNT membrane was transferred to one side of the sapphire substrate. The opposite side of the substrate was affixed to the ceramic heater using ceramic adhesive (Fig. 3a). The sample temperature was measured using a k-type thermocouple positioned in the immediate vicinity of the substrate. The SWCNT membrane was irradiated with collimated light from a halogen lamp. The transmitted light was focused onto the fibers and then measured using a spectrometer equipped with an indium gallium arsenide (InGaAs) array detector (NIRQuest + , Ocean Optics).

### Thermal radiation measurement

The membrane was transferred to the hollow honeycomb structure of the tungsten plate, forming a free-standing SWCNT membrane (Fig. 4a). This sample was thermally treated to decompose the polymers. First, the polymers wrapped around the SWCNTs were decomposed through a rapid annealing-and-cooling procedure^54^. During this procedure, the sample was placed in a quartz tube, pressurized at 5–10 Pa, and rapidly heated to 600 °C in a furnace. The sample was maintained at 600 °C for 5 min and then cooled to room temperature over a 10-min period under an argon flow. Thereafter, the sample was placed in a vacuum chamber, and an electric current was applied to the tungsten plate. The sample was heated by the Joule heat generated within the hollow honeycomb structure of the plate. The tungsten plate was cooled using copper blocks connected to liquid nitrogen. The thermal radiation of the SWCNT membrane was detected through a 600-μm diameter optical fiber placed in proximity to the SWCNT membrane (Fig. 4b) and then measured by the NIRQuest+ spectrometer equipped with the InGaAs array detector. The numerical aperture was increased (as much as possible) to prevent the entry of thermal radiation from the hotter tungsten plate into the fiber.

### Simulation of temperature distribution

The temperature distribution of a free-standing SWCNT membrane heated by the tungsten heater (Fig. 4a) was simulated using the heat-transfer module of COMSOL Multiphysics. Initially, we calculated the temperature distribution of the tungsten heater alone during current heating using a three-dimensional model of the heater. Supplementary Fig. 4a shows the simulation result. Here, the input current value was adjusted so that the edges of the central hexagonal part, which corresponds to the measurement area, reached 850 K. Each side of the central hexagon has a nearly uniform temperature of ≈850 K. Thus, when a free-standing SWCNT membrane is put on the heater, the membrane is expected to have a uniform temperature at its edges. Subsequently, we calculated the temperature distribution for the hexagonal SWCNT membrane, setting the boundary condition of its six sides to 850 K. The calculation for the membrane was performed using a two-dimensional model. For the thermal conductivity of the membrane, we used a typical value (30.4 W m^−1^ K^−1^) of SWCNT membranes reported in ref. ^55^. The emissivity of the SWCNT membrane at 850 K is deduced from the experimental results shown in Fig. 5a. Supplementary Fig. 4b–f shows the results after 1–10 s, indicating that the temperature of the SWCNT membrane within the central hexagonal part of the heater reaches 850 K after 10 s with a nearly uniform temperature distribution.

### Temperature estimation from the emissivity spectra

The temperature of the thermally emitting SWCNT membrane (Fig. 4) was determined from the spectral shape of the emissivity. We initially examined the absorbance spectra of the SWCNT membrane, which are consistent with the emissivity spectra according to Kirchhoff’s law^56^. Supplementary Fig. 5a shows the absorptance spectra of the free-standing SWCNT membrane at varying temperatures. These spectra were calculated using the optical transfer-matrix technique^52^, where the complex dielectric function was obtained from the on-sapphire membrane (Fig. 3d). Although the exciton peak was redshifted with temperature, the shapes of the absorptance spectra were less influenced by temperature than the complex dielectric function (Fig. 3d). After normalizing the absorbance spectra at different temperatures to the exciton peak intensity (Supplementary Fig. 5b), the normalized intensity of the exciton phonon sideband around 1.3–1.4 eV was ≈ 0.6 at all temperatures. Such spectral shape features of the emissivity spectra are expected from Kirchhoff’s law. Candidate emissivity spectra at various temperatures were calculated from the thermal radiation spectra. Supplementary Fig. 5c shows the resulting emissivity spectra with their intensities normalized at the *S*_11_ exciton resonance (1.1–1.2 eV). The normalized absorptance of the phonon sideband is largely temperature dependent and is ≈ 0.6 when taking the emissivity at an assumed temperature of 850 K. Supplementary Fig. 5d compares the emissivity spectra at an assumed temperature of 850 K (Supplementary Fig. 5c) with the absorptances at 800 K (Supplementary Fig. 5b). To account for the 50 K temperature difference, the absorptance spectra were shifted by 5 meV to lower photon energies. The 5-meV shift was determined by linearly extrapolating the temperature dependence of the absorptance peak of the *S*_11_ exciton (Supplementary Fig. 5d, inset) obtained from Supplementary Fig. 5a. Given the strong agreement between the two spectra, the temperature of the thermally emitting free-standing SWCNT membrane (Fig. 4c) was estimated as ≈ 850 K.

### Calculation of spectral selectivity

The emissivity spectrum of the SWCNT membrane was extrapolated to the lower photon-energy range using the tail of the Lorentz function to decrease the emissivity toward lower energies because the exciton oscillator intensity is preserved (Fig. 3h) and the excitons do not dissociate into free carriers.

### Few-layer architecture of SWCNT and MgO layers

We designed the planar few-layer architecture consisting of alternating SWCNT and MgO layers to enhance emissivity at the exciton resonance at 850 K. The emissivity was calculated using the optical transfer matrix method while varying the thickness of each layer^50^. The complex refractive index spectrum of MgO was taken from ref. ^53^. The designed structure was fabricated on an MgO substrate by alternating the transfer of SWCNT membranes and the deposition of MgO via electron beam evaporation^50^. The layer thicknesses of the fabricated structure were deduced from reflectance and transmittance measurements (Supplementary Fig. 7). The temperature dependence of the transmittance spectra of the few-layer architecture was measured using the setup shown in Fig. 3a. The thermal stability of the fabricated structure was confirmed (Supplementary Fig. 8), and the emissivity spectrum at 850 K was determined from the corresponding transmittance spectrum, following the same procedure used for the temperature-dependent optical susceptibility shown in Fig. 3d (Supplementary Fig. 9).

### (10,3) SWCNT refractive index

The complex refractive index spectrum of the (10,3) membrane at 1200 K was predicted based on the reported empirical spectrum at room temperature^30^, accounting for the temperature-induced shift in the exciton resonance energy expressed by $\Delta \hbar {\omega }_{0}(T)=-{A}_{0}{T}^{2}/(T+{T}_{0})$ (with ${A}_{0}$ = 0.179 meV K^−1^ and ${T}_{0}$ = 1800 K)^51^, and thermal broadening of the linewidth expressed by ${\hbar \gamma }_{{{\rm{L}}}}^{i}(T)={\hbar \gamma }_{{{\rm{L}}}0}^{i}+AT+B{(\exp ({T}_{0}/T)-1)}^{-1}$ (with ${\hbar \gamma }_{{{\rm{L}}}0}^{i}$ = 68.7 meV, A = 0.019 meV K^−1^, and B = 367 meV) for the *S*_11_ exciton and its phonon sideband, respectively. Meanwhile, their oscillator strengths are kept constant (similar results are shown in Fig. 3e–h), and other contributions are retained without modification.

### Schematics

Figures for the carbon nanotube structures were prepared with VESTA 3^57^, while further editing and other schematics were completed using Adobe Illustrator.

### Reporting summary

Further information on research design is available in the Nature Portfolio Reporting Summary linked to this article.

## Supplementary information

Supplementary Information

Reporting Summary

Transparent Peer Review file

## Source data

Source Data

## Data Availability

The data that support the findings of this study are available from the corresponding authors upon request. Source data are provided with this paper.
