# Supplementary material for: Exciton thermal radiation from macroscale membranes composed of chirality-sorted carbon nanotubes and its control
Source: Nat Commun. 2026 Jul 29;17:6967. doi: 10.1038/s41467-026-75711-4 (PMC13421534; doi:10.1038/s41467-026-75711-4)
Supplement: Supplementary file 1 — Supplementary Information [file 41467_2026_75711_MOESM1_ESM.pdf]

Supplementary Information

Exciton thermal radiation from macroscale membranes composed of chirality-sorted carbon nanotubes and its control

Akiteru Takahashi[1], Mioko Hizukuri[1,2], Kaichi Teranishi[1], Shonosuke Takaichi[1], Taishi Nishihara[1,3,4]*, Yuhei Miyauchi[1,2]*

[1]*Institute of Advanced Energy, Kyoto University, Kyoto 611-0011, Japan*

[2]*Department of Mechanical Engineering, The University of Tokyo, 7-3-1 Hongo, Bunkyo-ku, Tokyo 113-8656, Japan*

[3]*Department of Physics, Tokyo University of Science, Kagurazaka 1-3, Shinjuku, Tokyo 162-8601, Japan*

[4]*Research Institute for Science and Technology, Tokyo University of Science, Shinjuku, Tokyo 162-8601, Japan*

*Correspondence to: nishitai@rs.tus.ac.jp (T. N.), ymiyauchi@g.ecc.u-tokyo.ac.jp (Y. M.)

These authors contributed equally: Akiteru Takahashi, Mioko Hizukuri, Kaichi Teranishi

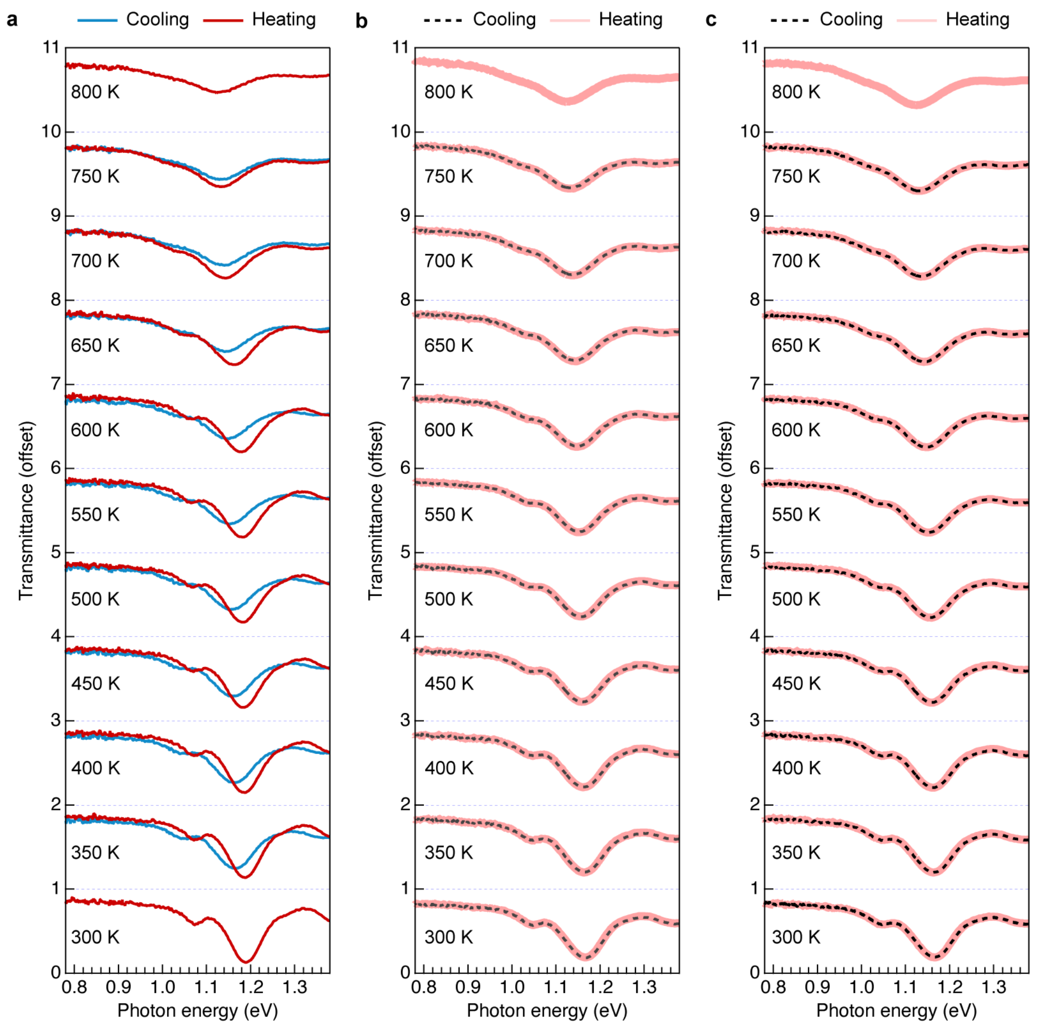

Supplementary Fig. 1. Temperature dependences of the transmittance spectra. The transmittance spectra of the (7,5) carbon nanotube membrane are shown during the **a** first, **b** second, and **c** third heating (from 300 to 800 K) and cooling (from 800 to 300 K) processes. The spectra are offset for visualization purposes (the dotted line in each spectrum indicates the zero line). Source Data are provided with this paper.

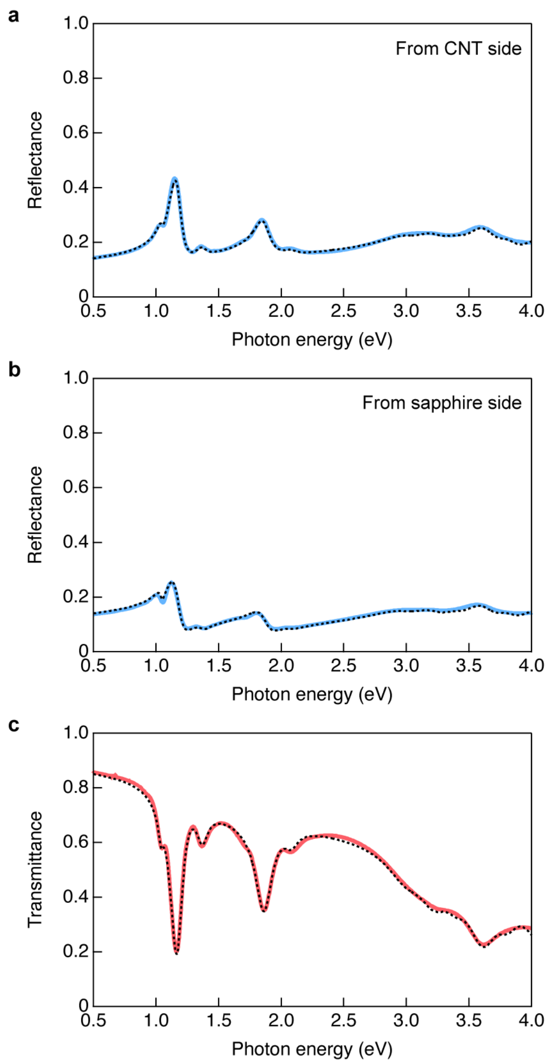

Supplementary Fig. 2. Optical spectra of the carbon nanotubes after the heating–cooling cycles. **a, b** Reflectance and **c** transmittance spectra of the (7,5) carbon nanotube (CNT) membrane after three heating–cooling cycles. The reflectance spectra are obtained in two configurations: **a** incident light entering from the CNT membrane side and **b** incident light entering from the sapphire substrate. The dotted black curves are the fitting results. Source Data are provided with this paper.

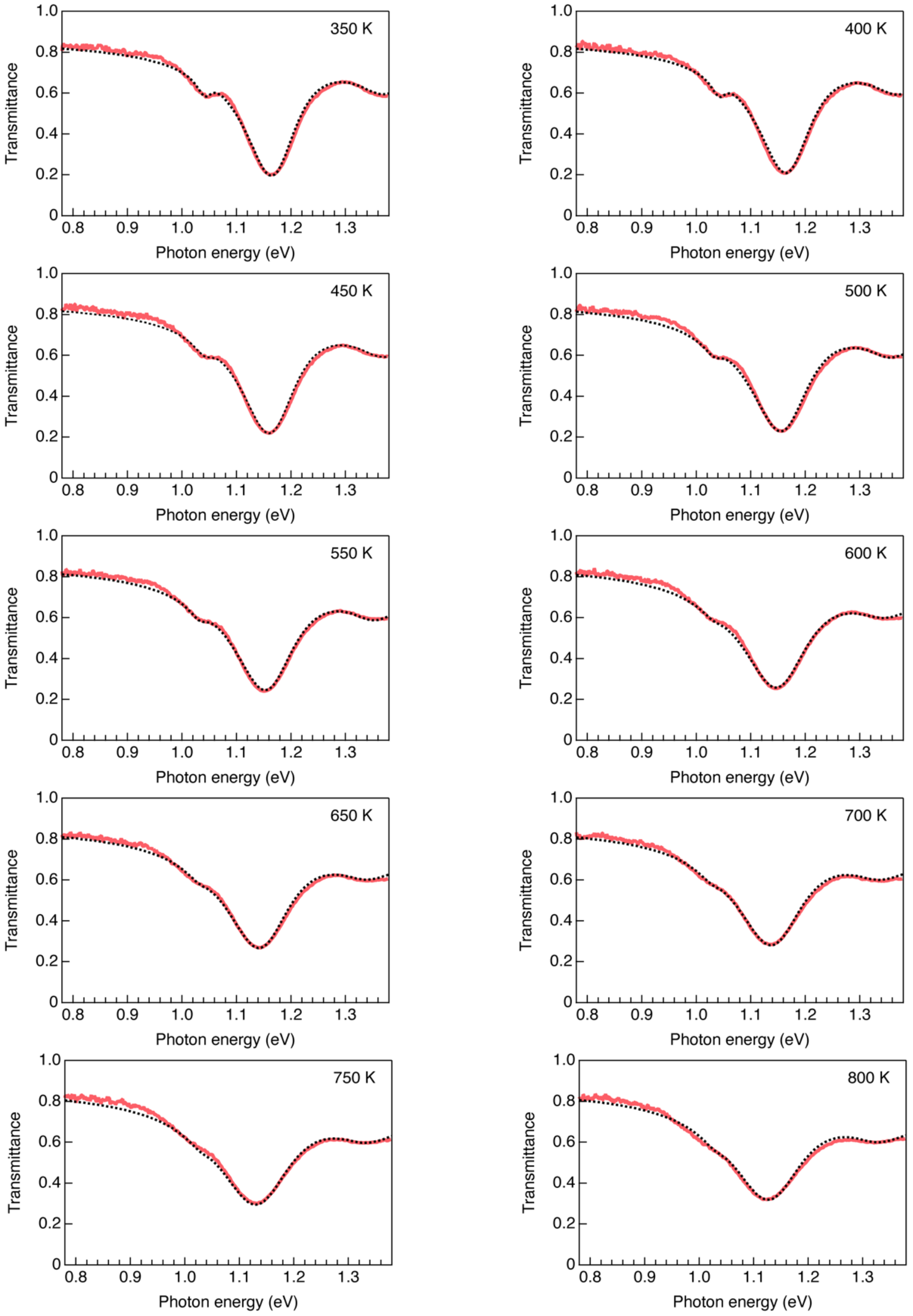

Supplementary Fig. 3. Transmittance spectra and their fitting results. The transmittance spectra (red curves) of the (7,5) CNT membrane are shown during the third cooling process obtained at different heating temperatures (350–800 K). The black dotted curves are the fitting results. Source Data are provided with this paper.

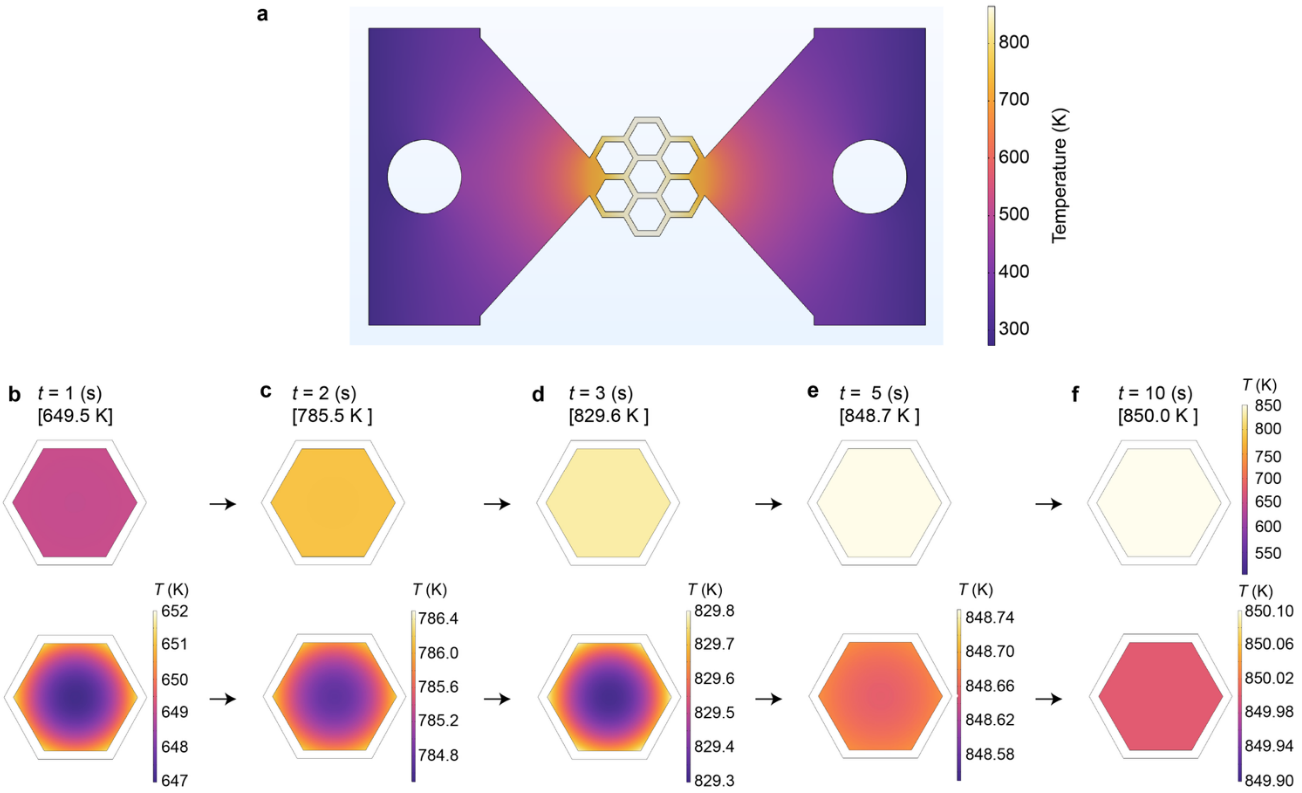

Supplementary Fig. 4. Simulated temperature distribution in a free-standing carbon nanotube membrane. a Temperature distribution in the designed tungsten heater. **b-f** Time-dependent temperature distribution in the carbon nanotube membrane. The upper panels are shown with a common color bar for comparison, while the lower panels use individual color bars to highlight the internal temperature distribution within each membrane at each time point.

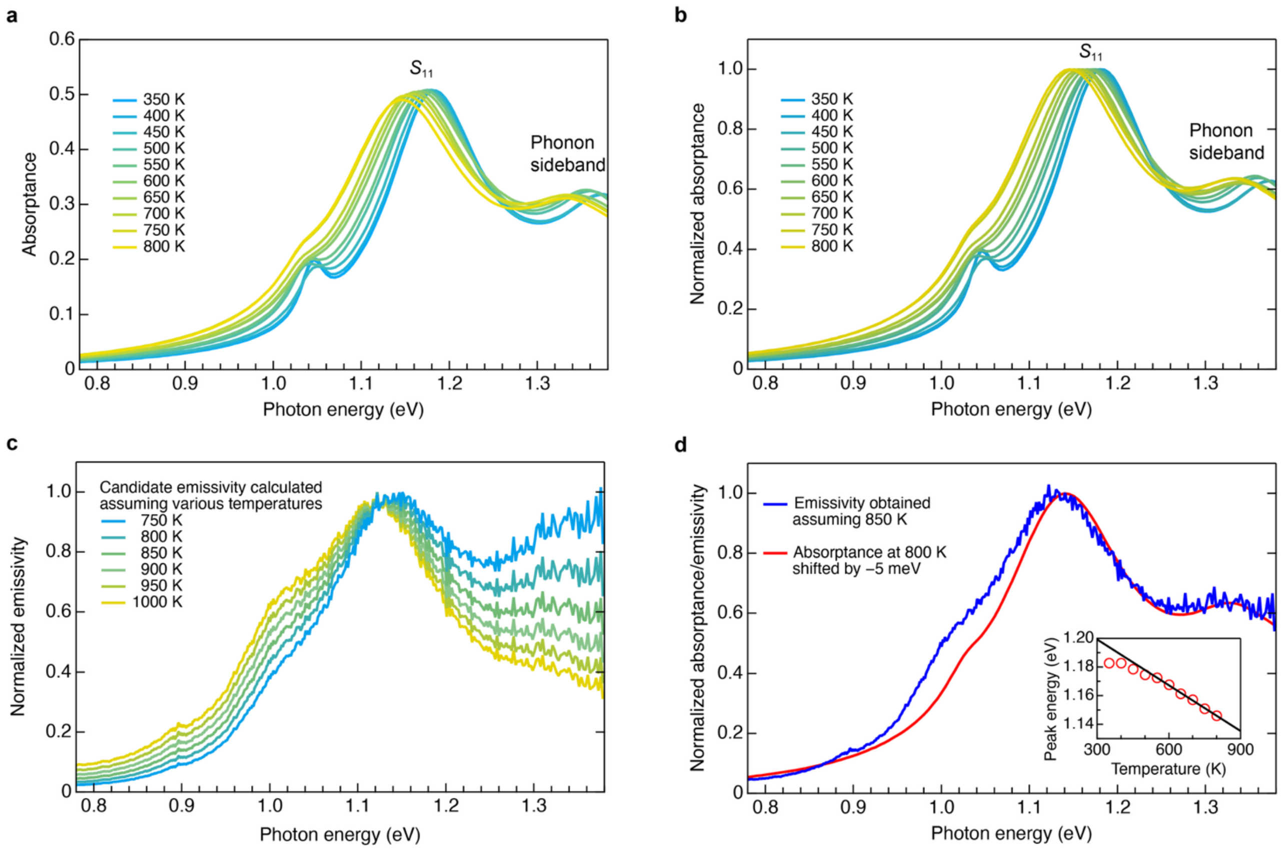

Supplementary Fig. 5. Temperature estimation from the absorptance and emissivity spectra. a Absorptance spectra of the free-standing SWCNT membrane at different temperatures calculated from the complex dielectric function (Fig. 3). **b** Absorptance spectra of the free-standing SWCNT membrane normalized at the absorptance intensity of the first subband (S_{11}) exciton. **c** Candidate emissivity spectra of the free-standing SWCNT membrane emitting thermal radiation (Fig. 4c) calculated assuming various temperatures (all intensities are normalized at the S_{11} exciton resonance 1.1–1.2 eV). As discussed in the main text, the only physically reasonable spectral emissivity is that at 850 K, while the others are not. **d** Comparison of the emissivity spectra obtained at an assumed temperature of 850 K and the absorbance at 800 K (left-shifted by 5 meV to correct for the 50 K temperature difference). The inset plots the temperature dependence of the absorbance peak of the S_{11} exciton (from panel **a**) and its linear fitting result (black line). Source Data are provided with this paper.

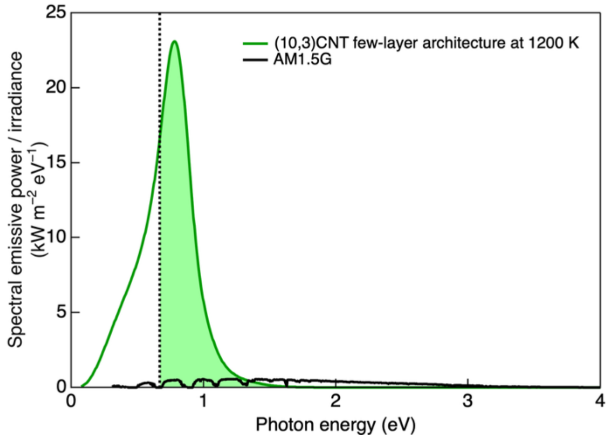

Supplementary Fig. 6. Spectral comparison. Solar spectrum (AM1.5G) and thermal radiation spectrum of a (10,3) carbon nanotube (CNT) few-layer architecture at 1200 K. Source Data are provided with this paper.

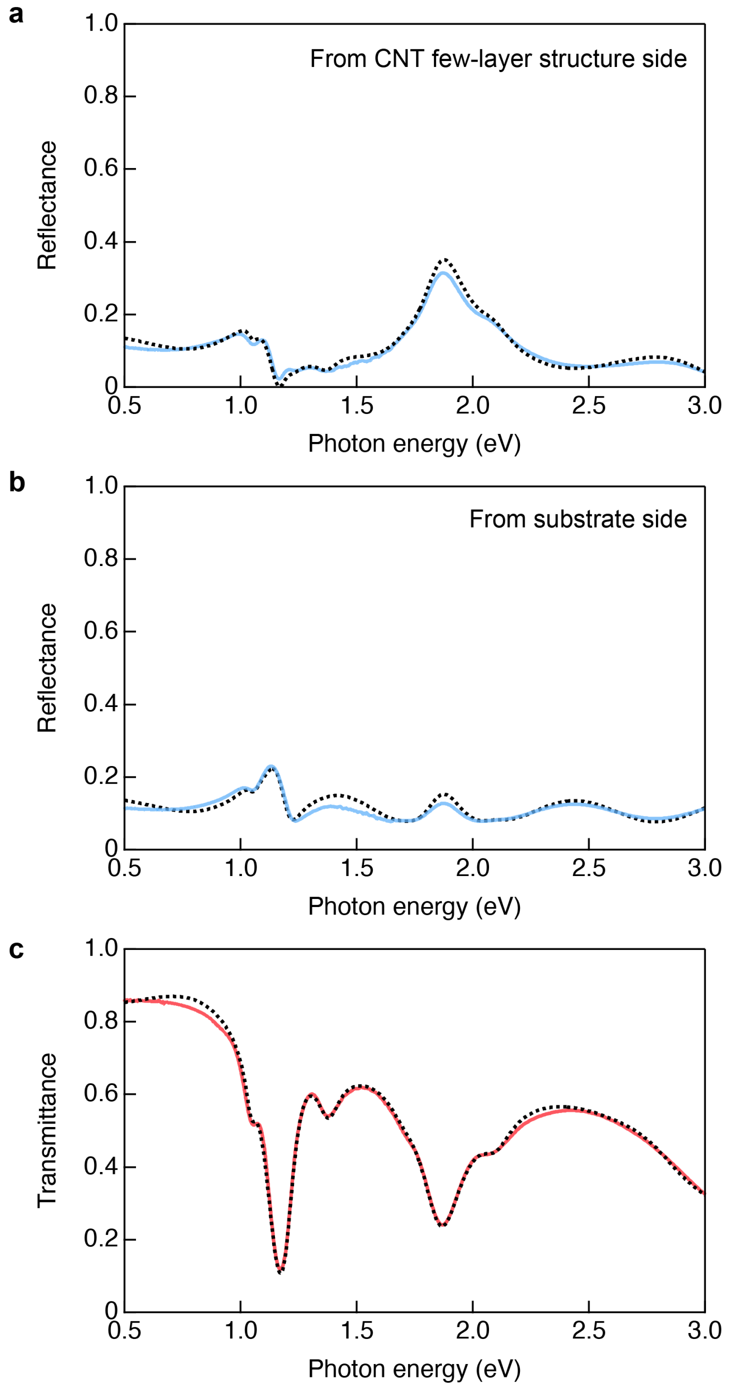

Supplementary Fig. 7. Optical spectra of carbon nanotube few-layer structure. a–c Reflectance (**a**,**b**) and transmittance spectra (**c**). The reflectance spectra are obtained in two configurations: incident light entering from the membrane side (**a**) and incident light entering from the MgO substrate (**b**). The black dotted curves are the fitting results. Source Data are provided with this paper.

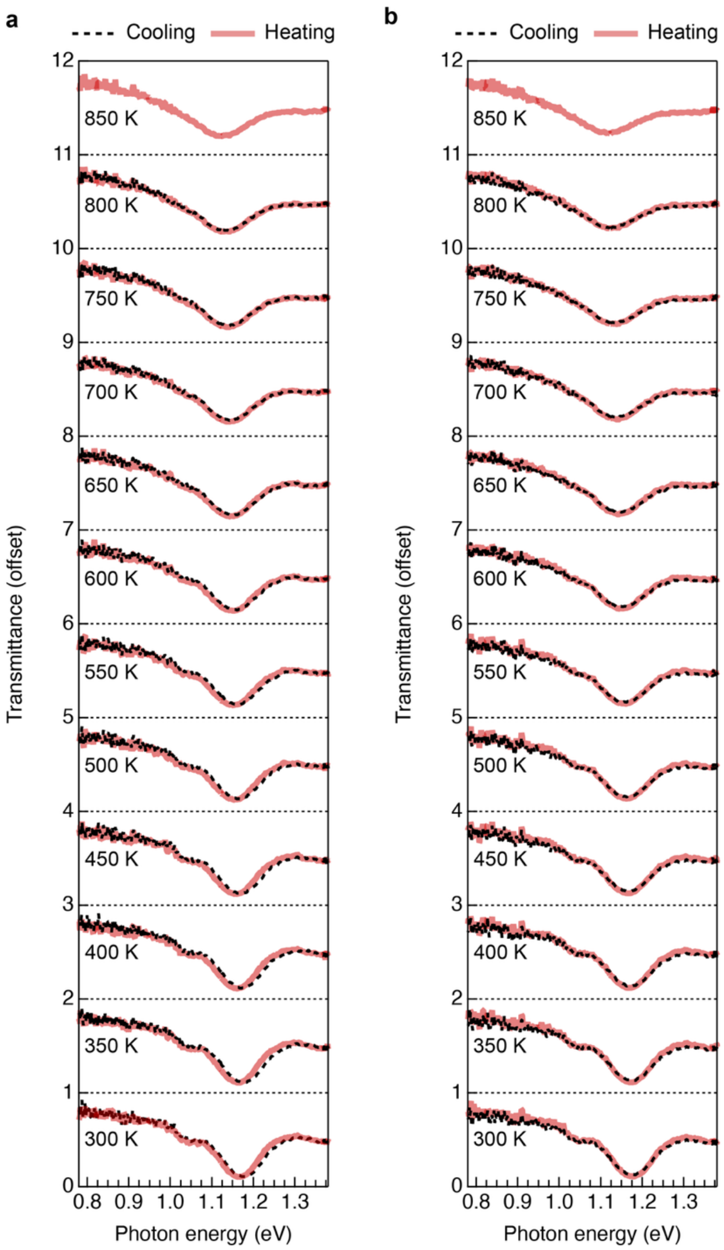

Supplementary Fig. 8. Temperature dependences of the transmittance spectra of the carbon nanotube few-layer structure. The transmittance spectra are shown during the **a** first and **b** second heating (from 300 to 850 K) and cooling (from 850 to 300 K) processes. The spectra are offset for visualization purposes (the dotted line in each spectrum indicates the zero line). Source Data are provided with this paper.

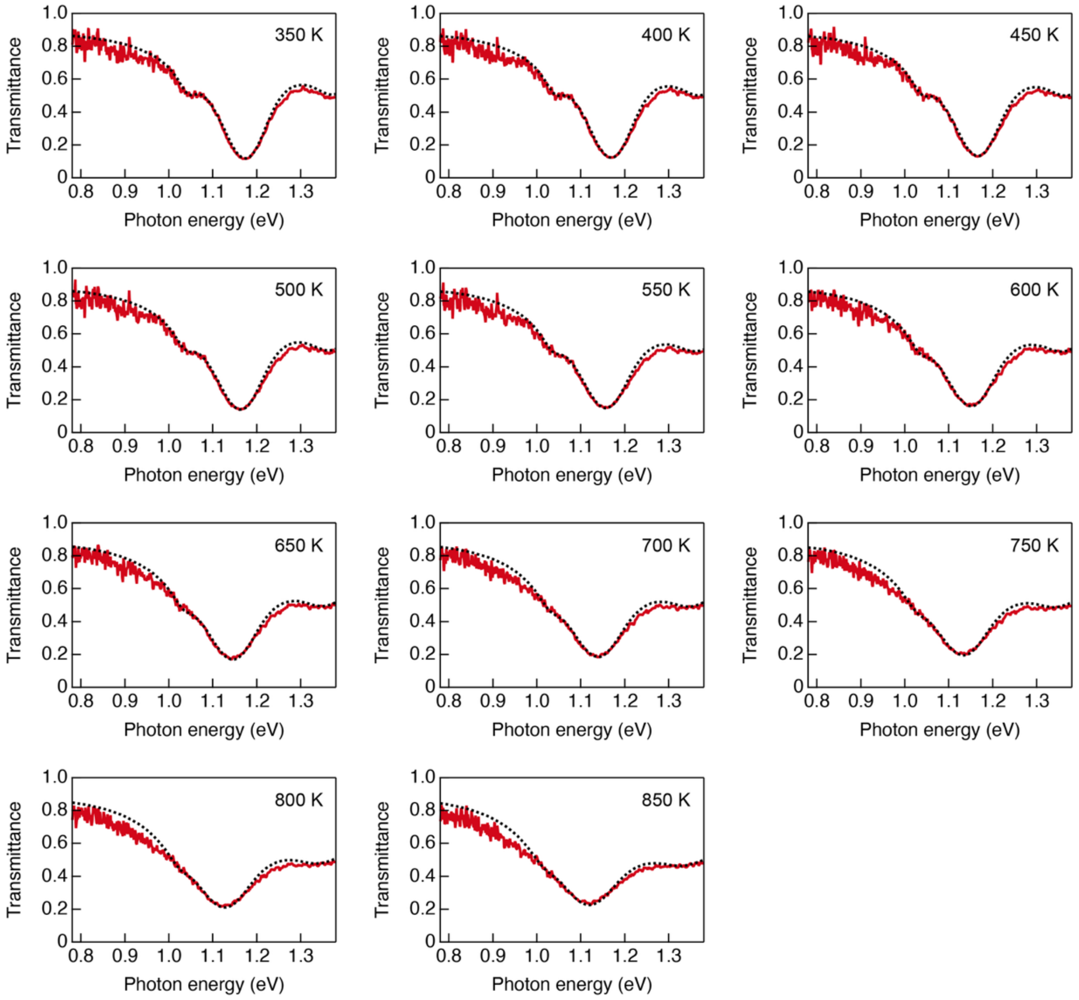

Supplementary Fig. 9. Transmittance spectra and their fitting results of the carbon nanotube few-layer structure. The transmittance spectra (red curves) are shown during the second heating process obtained at different heating temperatures (350–850 K). The black dotted curves are the fitting results. Source Data are provided with this paper.
