# Supplementary material for: Exciton thermal radiation from macroscale membranes composed of chirality-sorted carbon nanotubes and its control
Source: Nat Commun. 2026 Jul 29;17:6967. doi: 10.1038/s41467-026-75711-4 (PMC13421534; doi:10.1038/s41467-026-75711-4)
Supplement: Supplementary file 3 — Transparent Peer Review file [file 41467_2026_75711_MOESM3_ESM.pdf]

nature portfolio

Peer Review File

Exciton thermal radiation from macroscale membranes composed of chirality-sorted carbon nanotubes and its control

Corresponding Author: Professor Yuhei Miyauchi

This file contains all reviewer reports in order by version, followed by all author rebuttals in order by version.

Version 0:

Reviewer comments:

Reviewer #1

(Remarks to the Author)
It was a rare pleasure reading this manuscript that reports original, important results reached with a sound methodology and explained in a consistent, clear way.
The noteworthy result is the identification of excitonic peaks in the high-temperature emission spectra of single-chirality carbon nanotubes. This result is not only of general significance, adding substantially to the knowledge of optical properties of carbon nanotubes, but has also important practical consequences in applications of macroscopic nanotube samples as near-infrared emitters. The samples are of high quality, prepared in a very careful way, the measurements are exceptionally performed, and the results and their significance explained in depth for the expert but understandable for the general readership.
I fully endorse publication of this paper "as is" in Nature Communications.

Reviewer #2

(Remarks to the Author)
This manuscript reports on the thermal emission from excitons in single-wall carbon nanotubes (SWCNT) membranes. The authors prepare thin films of SWCNTs and measure both transmission and thermal emission as a function of temperature. Transmission measurements are used to extract the temperature dependence of the of the SWCNT array, and the spectral position and broadening of the SWCNT exciton resonance. From this data, the authors are able to construct a (temperature-dependent) permittivity for the SWCNT array, from which they are able to model thermal emission as a function of membrane temperature. They authors then heat the membrane by driving a current through it, and measure thermal emission from the heated membrane.
The thermal emission from the SWCNT membrane shows clear signatures of the exciton peaks for (7,5) and (7,6) SWCNTs, thus this work is able to demonstrate, and isolate, SWCNT thermal emission from excitons.
This is thorough work, and the results seem sound. Experimental results support the authors claims.
The authors have already published demonstration of high-temperature exciton emission in CNTs, and numerous other groups have investigated thermal emission from these structures. The results of this manuscript, given the authors' previous work on high temperature thermal emission from SWCNTs is not unexpected. The thermal emission itself, which, while narrower than a blackbody, is still rather broad. Integrating such structures into a thermovoltaic (TPV) system would require a very different architecture than what is presented in this work (of course, in this manuscript the authors are investigating the fundamental properties of the SWCNT membranes), and it is not clear that such structures would give the sort of performance necessary for actual TPV applications.

Reviewer #3

(Remarks to the Author)
The manuscript by Takahashi et. al. presented the first demonstration of thermal radiation from excitons in macroscopically assembled sorted carbon nanotubes (CNTs). The authors measured optical absorbance and thermal radiation at high temperatures (e.g., 850K) and observed clear exciton features in these spectra. Based on their prior works (Ref. 30), the authors also extracted optical constants of carbon nanotubes (CNTs) at various temperatures. Overall, the presented results

appear interesting. I have a few technical questions to be addressed before I can reach a conclusion.

(1) Regarding Fig. 1b, I don't quite understand the nature of "thermal excitation", which was achieved through direct thermal heating in experiments. Even for a temperature of 1000 K, the characteristic energy scale (< 100 meV) would be much smaller than the band gap of the used CNTs (~ 1 eV). How does this heating include the up transition depicted in Fig. 1b? Meanwhile, if any highly energetic process induces such a transition, why does the same process induce the dissociation of excitons, which only have the binding energy ~ 100s of meV (smaller than the bandgap)?

(2) Regarding Fig. 1c, the comparison between blackbody radiation and exciton thermal radiation appears misleading. Based on the current plot, it appears the intensity of exciton radiation from CNTs is comparable to blackbody radiation. But from experiments, the exciton radiation intensity is quite smaller than the blackbody radiation.

(3) Regarding the fitting of CNT dielectric constants, did the authors consider multiple reflections in CNT films or assume a thin-film approximation? Considering relatively large dielectric constants around excitons and CNT films, it could be possible that multiple reflection affects the fitting process.

(4) Regarding thermal radiation in SI Fig. 4c, what happens when the temperature is below 750K? The absorptance spectra in Fig. 4a span 350 to 800K instead.

(5) In addition, why is the (7,6) peak only observable at 1000K? Since the (7,5) exciton has a higher energy than the (7,6) and the (7,5) exciton peak is well resolved down to 750K, I expect the (7,6) peak to be also observable at lower temperature with a sharper linewidth. Can authors elaborate?

(6) Regarding the CNT film temperature estimate, I feel it would be better to cross-check the estimation, such as using a multiphysics simulation to simulate temperature profiles. The semiconducting CNT film is neither crystalline nor very conductive. The thermal conductance could be poor for the CNT film. The temperature may not be uniform inside a film.

(7) Finally, I am curious about the selective thermal emitter based on CNTs, which the authors have argued as a future application. If we look at Fig. 5, the CNT emission is also weaker than the blackbody radiation. Would it be better to construct a selective thermal emitter by putting a narrow-band near-infrared bandpass filter after a blackbody radiator, instead of using CNTs?

Version 1:

Reviewer comments:

Reviewer #2

(Remarks to the Author)
The authors largely addressed my concerns and I am able to recommend publication.

Reviewer #3

(Remarks to the Author)

I thank the authors for their detailed response. I still have a fundamental question:

(1) Regarding "thermally excited excitons" in Fig. 1b, following the authors' calculations, only a very tiny amount of thermally populated excitons is generated. Specifically, if the exciton level is ~1eV and T = 1000K, the exciton occupancy ratio exp(-E/kb*T) is ~ 0.001%. Then the thermal radiation from these excited excitons is intrinsically weak. I don't quite get how this exciton thermal emission in narrow-diameter CNTs can be useful for TPV applications around 1000 K.

If T = 1200K, the ratio becomes 0.006%, which is still quite small. The blackbody radiation peak is ~2.42 µm, corresponding to ~0.51 eV. However, getting larger-diameter CNTs with such a small bandgap seems still quite challenging, particularly for scalable film manufacturing.

Version 2:

Reviewer comments:

Reviewer #3

(Remarks to the Author)
The authors addressed my comment, and I don't have further comments.

Open Access This Peer Review File is licensed under a Creative Commons Attribution 4.0 International License, which permits use, sharing, adaptation, distribution and reproduction in any medium or format, as long as you give appropriate credit to the original author(s) and the source, provide a link to the Creative Commons license, and indicate if changes were made.
In cases where reviewers are anonymous, credit should be given to 'Anonymous Referee' and the source.
The images or other third party material in this Peer Review File are included in the article's Creative Commons license, unless indicated otherwise in a credit line to the material. If material is not included in the article's Creative Commons license and your intended use is not permitted by statutory regulation or exceeds the permitted use, you will need to obtain permission directly from the copyright holder.
To view a copy of this license, visit https://creativecommons.org/licenses/by/4.0/

Responses to the Reviewers' comments and suggestions

We are grateful for the reviewers' constructive comments and suggestions for improving the manuscript. We believe that these revisions and clarifications fully address all the reviewers' concerns and that the manuscript is now strengthened and suitable for publication in *Nature Communications*. Based on the reviewers' insightful feedback, we have carefully revised the manuscript. In the following, we present point-by-point responses to the reviewers' comments and the revisions included in the revised manuscript.

Summary of major revisions

- To prevent misunderstandings regarding the heating method and to ensure it is unambiguously clear that the sample was heated by thermal conduction, the relevant expressions in the main text have been revised.
- Addition of new experimental data (Fig. 6) according to Q2-3) and Q3-7): Demonstration of emissivity enhancement in an all-dielectric SWCNT–MgO few layer architecture, maintaining performance at elevated temperatures up to 850 K.
- Addition of a discussion and a figure (Fig. 7) on the roadmap for actual TPV applications (Page 12, 2nd paragraph) according to Q2-3) and Q3-7): Proposal of using thermally-stable larger-diameter CNTs combined with the proposed few-layer structure, which yields a very high spectral efficiency of 0.64 at 1200 K, significantly exceeding the value of black body emitter (0.12) at the same temperature.
- Multiphysics simulations using COMSOL Multiphysics (Supplementary Fig. 4) according to Q3-6): Verification of nearly uniform temperature distribution within the free-standing SWCNT membrane, ensuring the reliability of the thermal emission measurements.

Summary of other revisions

- We added the author who conducted additional emitter design and experiments.
- In the abstract, we added some sentences.
- On page 5 (6th line from bottom of the 1st paragraph), we added sentences.
- In the Methods, we added some sections.
- In Fig. 1, we revised panel **c** and the corresponding caption according to Q3-2).
- In Supplementary Fig. 5, we added sentences to panel **c** and the corresponding caption according to Q3-4).
- We added Supplementary Figs. 6, 7 and 8 in connection with Q2-3) and Q3-7).

For Reviewer #1:

It was a rare pleasure reading this manuscript that reports original, important results reached with a sound methodology and explained in a consistent, clear way. The noteworthy result is the identification of excitonic peaks in the high-temperature emission spectra of single-chirality carbon nanotubes. This result is not only of general significance, adding substantially to the knowledge of optical properties of carbon nanotubes, but has also important practical consequences in applications of macroscopic nanotube samples as near-infrared emitters. The samples are of high quality, prepared in a very careful way, the measurements are exceptionally performed, and the results and their significance explained in depth for the expert but understandable for the general readership. I fully endorse publication of this paper "as is" in Nature Communications.

We thank the Reviewer for their positive evaluation of our work and for recommending it for publication in Nature Communications.

For Reviewer #2:

This manuscript reports on the thermal emission from excitons in single-wall carbon nanotubes (SWCNT) membranes. The authors prepare thin films of SWCNTs and measure both transmission and thermal emission as a function of temperature. Transmission measurements are used to extract the temperature dependence of the SWCNT array, and the spectral position and broadening of the SWCNT exciton resonance. From this data, the authors are able to construct a (temperature-dependent) permittivity for the SWCNT array, from which they are able to model thermal emission as a function of membrane temperature. They authors then heat the membrane by driving a current through it, and measure thermal emission from the heated membrane. The thermal emission from the SWCNT membrane shows clear signatures of the exciton peaks for (7,5) and (7,6) SWCNTs, thus this work is able to demonstrate, and isolate, SWCNT thermal emission from excitons. This is thorough work, and the results seem sound. Experimental results support the authors claims.

We thank the Reviewer for evaluating our work as "thorough" and "sound". Since we believe that there is a factual inaccuracy regarding our experimental methodology in the Reviewer's summary above, first we would like to point it out. Specifically, the Reviewer stated that "*They authors then heat the membrane by driving a current through it, and measure thermal emission from the heated membrane,*" whereas, in reality, we did not drive any electric current through the carbon nanotube (CNT) membrane, but rather heated it via thermal conduction. We regret that the ambiguity in the previous manuscript's wording led to misunderstandings, and the relevant expressions in the main text have been revised to clarify that the sample was heated by thermal conduction. Therefore, we would be grateful if the Reviewer's assessment of this work could be reconsidered, having revised his/her understanding of this point.

Q2-1) The authors have already published demonstration of high-temperature exciton emission in CNTs, and numerous other groups have investigated thermal emission from these structures.

A2-1) As the Reviewer commented, our group has previously demonstrated both thermal and nonthermal excitonic radiation from "individual SWCNTs" under laser heating and electronic current injection conditions [Refs 9, 10], and other groups have also reported peaked thermal emission from "individual SWCNTs" by electric current injection heating [Refs 31, 32]. Thermal radiation from "ensembles composed of SWCNTs with variety of chiral structures" have also been reported [Refs 36–42], where only blackbody-like broadband emission, which is completely different from peaked excitonic thermal radiation observed for the membranes of structurally-pure SWCNTs in this study,

has been reported. However, we respectfully disagree with the reviewer's view because of the following reasons.

First, **no experiments similar to those conducted in this study have been reported to date.** As described above, we do not drive any electric current through the SWCNT membrane. As we have recently reported [T. Nishihara, Y. Miyauchi *et al.*, *Commun. Mater.* **6**, 89 (2025)], all the above heating methods (optical and electrical methods) potentially cause non-thermal excitation of excitons. Instead, we heat the SWCNT membrane via thermal conduction to ensure that local thermal equilibrium is reliably maintained within the SWCNTs.

The distinction in the heating method from previous studies is fundamental to the novelty of our work. While current injection heating (Joule heating) is only applicable to metallic or heavily carrier-doped semiconducting SWCNTs and their mixture, it is fundamentally inapplicable to the undoped, single-chirality sorted, intrinsic semiconducting SWCNT membranes used in our study. **Therefore, current injection heating of our semiconducting SWCNT membrane is fundamentally impossible**. To somehow apply that method to our sample, heavy carrier doping is necessary, but under carrier-doped conditions, the charge neutrality of the semiconducting SWCNTs is not maintained, which potentially results in completely different thermal emission behavior from that of intrinsic semiconducting SWCNTs because of the different conditions for dielectric screening and state-filling.

Furthermore, to the best of our knowledge, our observation of excitonic thermal radiation from an object with a macroscopic area via thermal conduction is the first report of its kind, not only for SWCNTs but for any material in the history of research into thermophotonic properties of materials. Even only by this fact, we believe it is clear that our results cannot be evaluated as being similar to previously reported results.

Q2-2) The results of this manuscript, given the authors' previous work on high temperature thermal emission from SWCNTs is not unexpected.

The previously reported fact by our group that individually isolated SWCNTs exhibit exciton thermal radiation does not guarantee that exciton thermal radiation can be observed when heating an aggregate composed of SWCNTs with a uniform structure. This is because, as shown in Fig. R1, aggregated SWCNTs experiences stronger dielectric screening than individually isolated SWCNTs, resulting in a lower exciton binding energy. Therefore, the results of this study, which demonstrated that exciton thermal radiation can indeed be observed in SWCNT assemblies through experimentation, are by no means self-evident, but rather significant because they open the possibility of utilizing thermally robust quantum effects in nanomaterials in macroscale energy technologies.

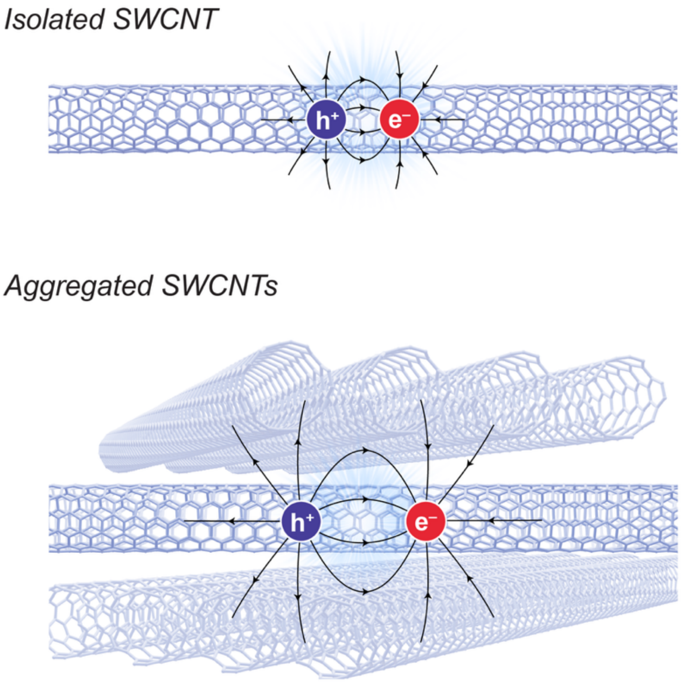

Fig. R1 | Schematic of an exciton in an isolated single-walled carbon nanotube (SWCNT; top panel) and aggregated ones (bottom panel). The black arrows represent the electric field between an electron (e^{-}) and a hole (h^{+}).

Q2-3) The thermal emission itself, which, while narrower than a blackbody, is still rather broad. Integrating such structures into a thermovoltaic (TPV) system would require a very different architecture than what is presented in this work (of course, in this manuscript the authors are investigating the fundamental properties of the SWCNT membranes), and it is not clear that such structures would give the sort of performance necessary for actual TPV applications.

A2-3) We agree with the Reviewer's assessment that further architectural and material optimization is essential for integrating structure-sorted SWCNT membranes into high-performance TPV systems. While the primary focus of this manuscript remains the fundamental characterization of the excitonic thermal radiation from structure-sorted SWCNT membranes, in the revised manuscript, we have provided a clear roadmap to bridge this gap. Specifically, we address the spectral band width and system performance through two strategies:

(1) Implementation of an all-dielectric SWCNT few-layer structure:

To overcome the theoretical emissivity limit (0.5) of 2D membranes, we have recently developed an all-dielectric few-layer planar structure. This structure utilizes optical interference to significantly

enhance absorptivity (emissivity) at the exciton resonance [M. Hizukuri *et al.*, *ACS Photonics* **12**, 6984 (2025)]. While our previous study focused on room temperature properties, in the revised manuscript, we have added new experimental results at high temperatures as Fig. 6 (this figure is reproduced below as Fig. R2). These data demonstrate that the (7,5) SWCNT–MgO few layer structure maintains enhanced absorptivity (emissivity) at elevated temperatures up to 850 K. This simple four-layer architecture enables high spectral peak intensity without the need for complex lateral lithographic patterning for photonic control, offering a significant advantage for large-scale TPV implementation.

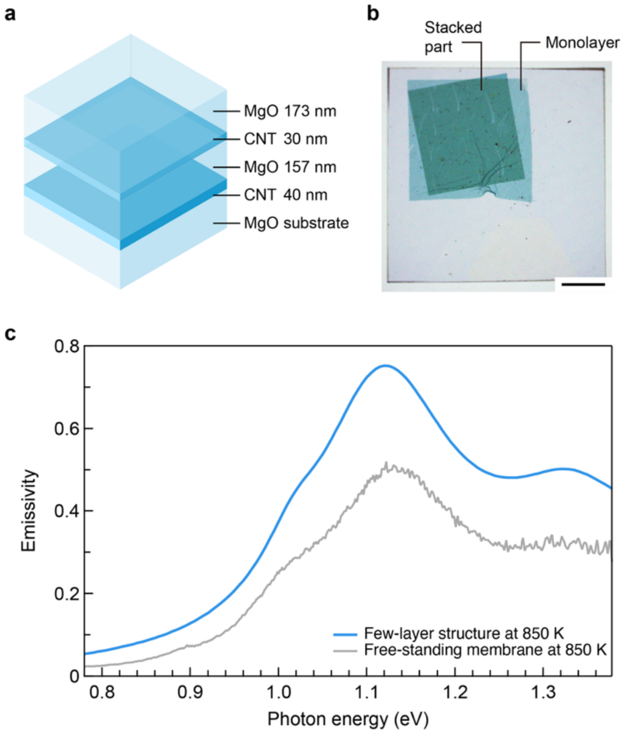

Fig. R2 | Carbon nanotube few-layer architecture. a,b A schematic (**a**) and a photograph (**b**) of the (7,5) carbon nanotube (CNT)–MgO few layer architecture (scale bar = 2 mm). **c** The emissivity spectra of the fabricated few-layer architecture (blue) and the free-standing membrane (gray) at 850 K. The spectrum of the few-layer architecture is obtained from its transmittance spectrum at 850 K. The spectrum of the free-standing membrane is the same as the one shown in Fig. 5a.

(2) Use of larger-diameter SWCNTs for high-temperature operation:

The Reviewer noted that the observed thermal radiation spectrum is still relatively broad. We agree with this comment, but we clarify that the apparent broadness at 850 K is primarily due to the large energy mismatch between the (7,5) SWCNTs' exciton resonance peak (~1.0 eV) and the peak of the Planck distribution peak at that temperature (~0.2 eV). In this regime, even with very low off-resonance emissivity, the relative proportion of infrared radiation remains high.

For practical TPV applications, operation at temperatures above ~1200 K is required to obtain sufficient photon flux (typically higher than the solar photon flux of ~1 kW m^{-2}) above the PV cell bandgap (e.g., 0.67 eV for GaSb). While (7,5) SWCNTs were used in this study as a model material for which relatively high-purity sample is readily available, their small diameter (~0.83 nm) introduces lattice strain that limits their thermal stability above 850 K. However, by employing larger diameter SWCNTs, we can simultaneously address both stability and spectral matching issues.

Firstly, as compared in Fig. R3a, larger diameter SWCNTs such as (10,3) SWCNTs with diameter of ~0.92 nm exhibit higher thermal stability due to lower strain. Our tests confirm that (10,3) SWCNT membranes maintain their optical properties even after treatment at 1173 K, as shown in Fig. R3b.

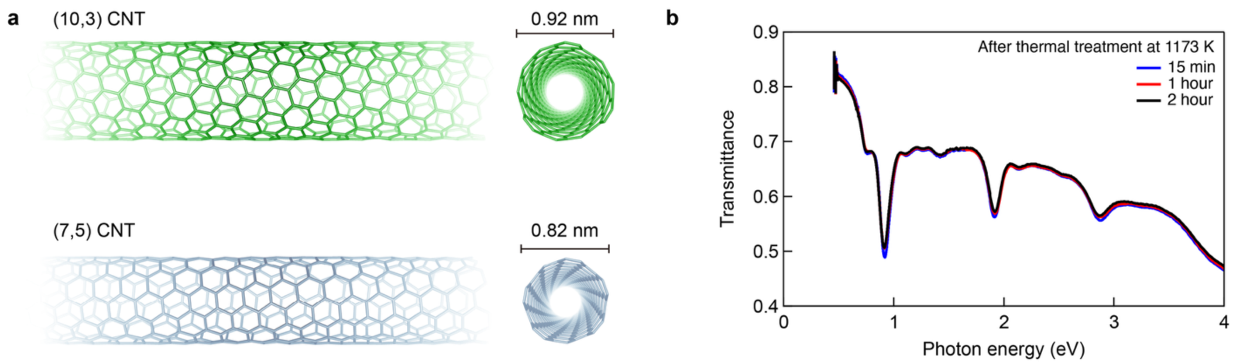

Fig. R3 | a Structures of (7,5) and (10,3) SWCNTs, illustrating the difference in tube diameter. The right panels display the corresponding cross-sectional views. **b** Thermal stability test of a (10,3) SWCNT membrane; the transmittance spectra show the retention of optical spectra after annealing at 1173 K.

Secondly, using larger diameter SWCNTs (here we adopt (10,3) SWCNTs as one example) shifts the exciton resonance to lower energy side. At ~1200 K, where the Planck distribution peak shifts to ~0.5 eV, this ensures a much higher thermal radiation intensity (~ 6 kW m^{-2}) than (7,5) SWCNT few-layer structure at 850 K (~ 0.04 kW m^{-2}) above the PV cell band gap, and much improves the spectral selectivity and PV energy conversion efficiency. Using the complex refractive index predicted from our previous studies [29] (Fig. R4a), we simulated the emissivity and thermal radiation spectrum of a (10,3) SWCNT–MgO few layer architecture (Fig. R4b) at 1200 K. The results of the emissivity (Fig. R4c) and thermal radiation spectra (Fig. R4d) demonstrate that the spectral efficiency, defined as the ratio of power emitted above the PV band gap (here, we set it as 0.67 eV for a typical GaSb cell) to total power, reaches 0.64. This is a five-fold increase over blackbody radiation (0.12), and exceeds values reported for various sophisticated photonic crystal/meta surface emitters in the literature at most less than 0.5 [S. Zhang *et al*., *Adv. Opt. Mater.* **12**, 2301726 (2024)].

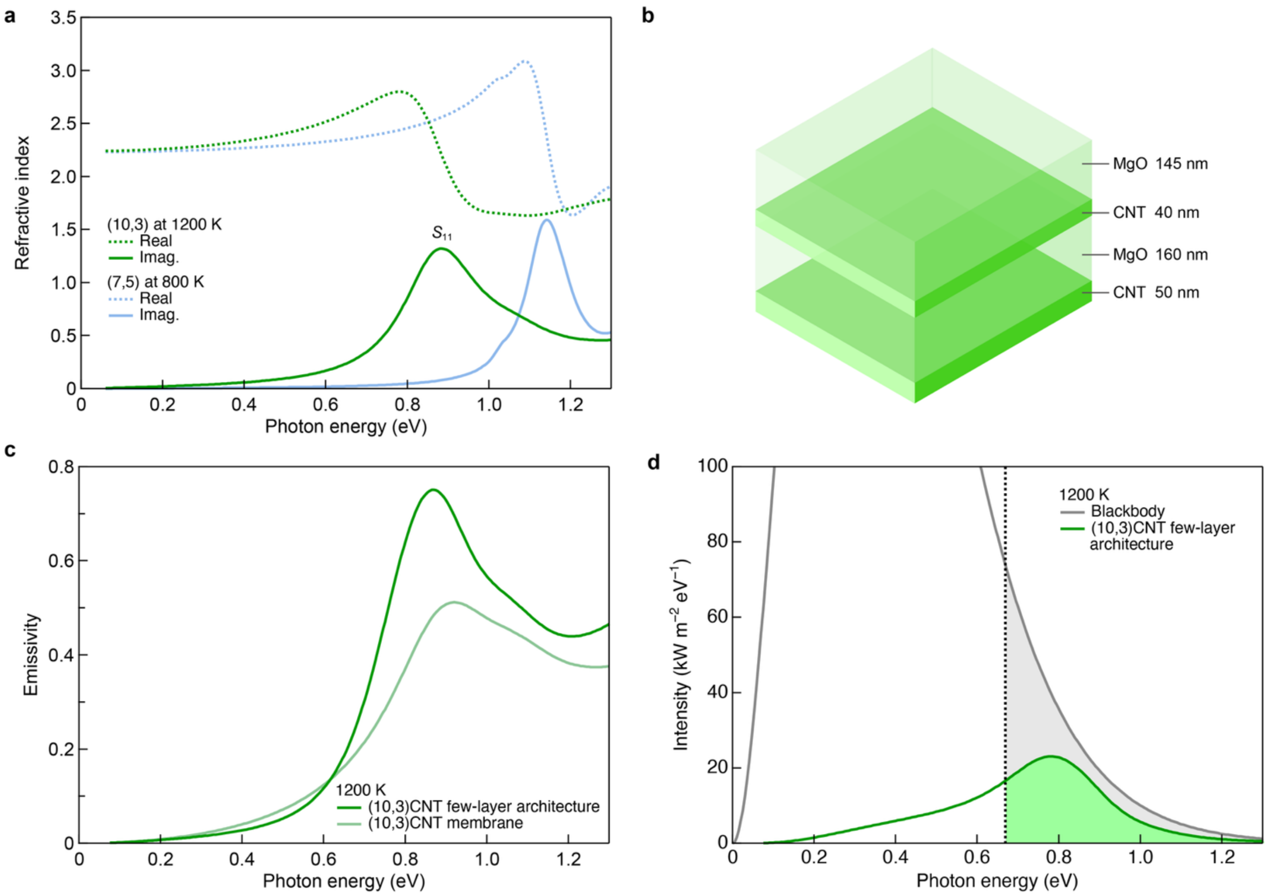

Fig. R4 | **a** Simulated complex refractive index spectra of (10,3) CNT membrane at 1200 K (solid curves), compared with the measured complex refractive index spectra of the (7,5) CNT membrane at 800 K (blue curves). **b** Schematic of the (10,3) CNT few-layer architecture. **c,d** Simulated emissivity spectrum (**c**) and thermal radiation spectra (**d**) of the CNT few-layer architecture at 1200 K. In **c**, the light green curve shows the emissivity spectrum of a single-layer membrane with a thickness of 90 nm. In **d**, the gray curve shows the blackbody radiation at 1200 K. The dotted line indicates a bandgap energy of typical GaSb photovoltaic cell of 0.67 eV.

While a full experimental demonstration of TPV power generation using larger diameter SWCNTs is beyond the scope of this study and being prepared for a separate publication, in summary, we have updated the revised manuscript as follows to clarify the above points:

1. We added Fig. 6 showing experimental results for the (7,5) SWCNT few-layer architecture at 850 K.
2. We updated the discussion (Page 11, 2nd paragraph) to include the design strategy for 1200 K operation using larger-diameter SWCNTs, supported by the simulation results mentioned above.

We believe that these additions clearly demonstrate the potential of our excitonic emitter platform for future TPV technology.

For Reviewer #3:

The manuscript by Takahashi et. al. presented the first demonstration of thermal radiation from excitons in macroscopically assembled sorted carbon nanotubes (CNTs). The authors measured optical absorbance and thermal radiation at high temperatures (e.g., 850K) and observed clear exciton features in these spectra. Based on their prior works (Ref. 30), the authors also extracted optical constants of carbon nanotubes (CNTs) at various temperatures. Overall, the presented results appear interesting. I have a few technical questions to be addressed before I can reach a conclusion.

We thank the reviewer for his/her evaluation of our paper as "interesting". According to the reviewer's insightful comments, we conducted additional simulations, and improved the manuscript. The point-by-point responses to the reviewer's concerns are shown below.

Q3-1) Regarding Fig. 1b, I don't quite understand the nature of "thermal excitation", which was achieved through direct thermal heating in experiments. Even for a temperature of 1000 K, the characteristic energy scale (< 100 meV) would be much smaller than the band gap of the used CNTs (~ 1 eV). How does this heating include the up transition depicted in Fig. 1b? Meanwhile, if any highly energetic process induces such a transition, why does the same process induce the dissociation of excitons, which only have the binding energy ~ 100s of meV (smaller than the bandgap)?

A3-1) The thermal excitation (population) of excitons is described by the Bose–Einstein distribution function of $f(T,\omega) = [e^{\hbar\omega/k_{\mathrm{B}}T} - 1]^{-1}$ (T: Temperature, ω: Optical frequency). While the average thermal energy $k_{\mathrm{B}}T$~90 meV at ~1000 K is indeed smaller than the exciton energy of ~1 eV, the thermal distribution has a finite tail at high energies. This allows for a finite steady-state population of excitons. Microscopically, excitons can be excited through multi-phonon absorption processes, as we previously clarified theoretically [S. Konabe *et al.*, *Opt. Lett.* **46**, 3021 (2021)]. Regarding the dissociation, the binding energy of E_{b}~0.26 eV (c.f. Fig. 1b for E_{b}) recently revealed by our group [Z. Liu *et al.*, *ACS Nano* **19**, 41252 (2025)] provides a significant energy barrier. The ratio of the distribution function at the continuum edge (quasiparticle band gap) (E_{b} ~1.26 eV) to the exciton level (~1 eV) is approximately $e^{-E_{\mathrm{b}}/k_{\mathrm{B}}T} \approx 0.05$ at 1000 K, confirming that the majority of thermally excited excitons remain stable and contribute to the distinct resonance peak.

Q3-2) Regarding Fig. 1c, the comparison between blackbody radiation and exciton thermal radiation appears misleading. Based on the current plot, it appears the intensity of exciton radiation from CNTs is comparable to blackbody radiation. But from experiments, the exciton radiation intensity is quite smaller than the blackbody radiation.

A3-2) We thank the reviewer for this important comment. As the reviewer pointed out, Fig. 1c in the previous manuscript could indeed be misleading. The intention of this figure was to show that the exciton thermal radiation has a narrower bandwidth compared to blackbody radiation. Therefore, in the revised manuscript, we have modified Fig. 1c to show the spectra normalized at the exciton resonance energy to facilitate a comparison of the linewidths. We have also added a sentence to the caption of Fig. 1 as "The peak intensity of exciton thermal radiation is normalized to blackbody radiation curve.".

Q3-3) Regarding the fitting of CNT dielectric constants, did the authors consider multiple reflections in CNT films or assume a thin-film approximation? Considering relatively large dielectric constants around excitons and CNT films, it could be possible that multiple reflection affects the fitting process.

A3-3) Yes, we have taken into account the multiple reflections in CNT films when determining dielectric spectra using fitting. The reflection and transmission spectra of CNT thin films on sapphire surfaces were calculated using the optical transfer matrix method, which accounts for multiple reflections [D. S. Bethune, *J. Opt. Soc. Am. B* **6**, 910 (1989)]. Furthermore, we have considered reflections and transmissions at the rear surface of the sapphire substrate [T. Nishihara *et al.*, *Nanophotonics* **11**, 1011, (2022)].

Q3-4) Regarding thermal radiation in SI Fig. 4c, what happens when the temperature is below 750K? The absorptance spectra in Fig. 4a span 350 to 800K instead.

A3-4) Supplementary Fig. 5c of the revised manuscript shows 'candidates for the emissivity spectra', but they are not the thermal radiation (emission) spectra themselves. Since it was difficult to measure the temperature of the free-standing CNT membrane directly, we determined the temperature based on the comparison of its emissivity spectral shape with that of the absorptivity determined by the other measurements. Supplementary Fig. 5c compares the candidates for the emissivity spectra which is calculated by dividing the measured thermal radiation spectrum by Planck's function of various temperatures from 750 K to 1000 K.

Figure R5 shows the resulting ‘candidates’ of the emissivity spectra obtained assuming temperatures below 750 K. Their shapes are significantly different from the predicted absorptivity spectra of the free standing SWCNT membrane (Supplementary Fig. 5a). Since the spectral emissivity should be consistent with the absorptivity according to Kirchhoff’s law, it is concluded that the radiation temperature is not below 750 K.

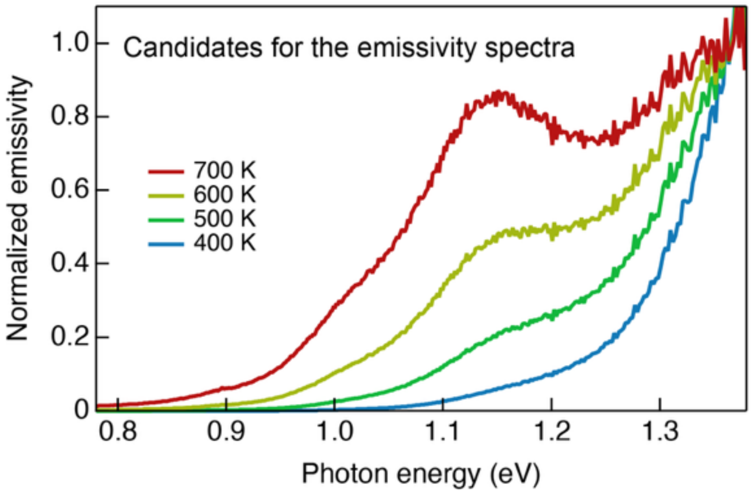

Fig. R5 | Candidate emissivity spectra of the free-standing CNT membrane emitting thermal radiation (Fig. 4c) assumed at temperatures below 750 K (all intensities are normalized at the absorptance at 1.38 eV).

The key characteristic of the absorptivity spectra (Supplementary Fig. 5a, 350–800 K) is that the phonon sideband feature at 1.3–1.4 eV is approximately 0.6 times the exciton absorptance regardless of the temperature. Among the candidate spectra in Supplementary Fig. 5c, only the spectrum corresponding to 850 K satisfies this characteristic. Therefore, we determined the radiation temperature of the CNT membrane to be 850 K.

To avoid readers’ confusion, we added a sentence in the caption of Supplementary Fig. 5c as “Candidate emissivity spectra … calculated assuming various temperatures”, and a sentence in the caption of Supplementary Fig. 5c as “As discussed in the main text, the only physically reasonable spectral emissivity is that at 850 K, while the others are not.”.

Q3-5) In addition, why is the (7,6) peak only observable at 1000K? Since the (7,5) exciton has a higher energy than the (7,6) and the (7,5) exciton peak is well resolved down to 750K, I expect the (7,6) peak to be also observable at lower temperature with a sharper linewidth. Can authors elaborate?

A3-5) As explained in A3-4) above, among the plots in Supplementary Fig. 5c, only the one for 850 K represents the true spectral emissivity spectrum. The spectrum calculated assuming 1000 K is the inappropriate one. The reason the (7,6) CNT peak is clearly visible in that plot is simply because the emissivity spectrum was calculated using an incorrect temperature of 1,000 K. As mentioned in A3-4), we added sentences in the Supplementary Fig. S5c and its caption to avoid readers' confusion.

As the reviewer pointed out and as shown in Supplementary Fig. 5a, the exciton resonance linewidth in the absorptivity spectrum sharpens at lower temperatures, allowing the (7,5) and (7,6) peaks to be better resolved. Figure R6 compares the thermal radiation spectrum, predicted using the 750 K absorptivity determined through the temperature-dependent transmittance measurements, with the measured radiation spectrum of the CNT membrane at 850 K. Since thermal radiation intensity increases exponentially with temperature, the radiation intensity at 750 K is significantly lower than that at 850 K. In addition, at 750 K, the radiation intensity at the photon energy below the exciton resonance energy is much stronger than that at the exciton energy, which makes it difficult to clearly resolve the (7,6) exciton thermal radiation peak from other features.

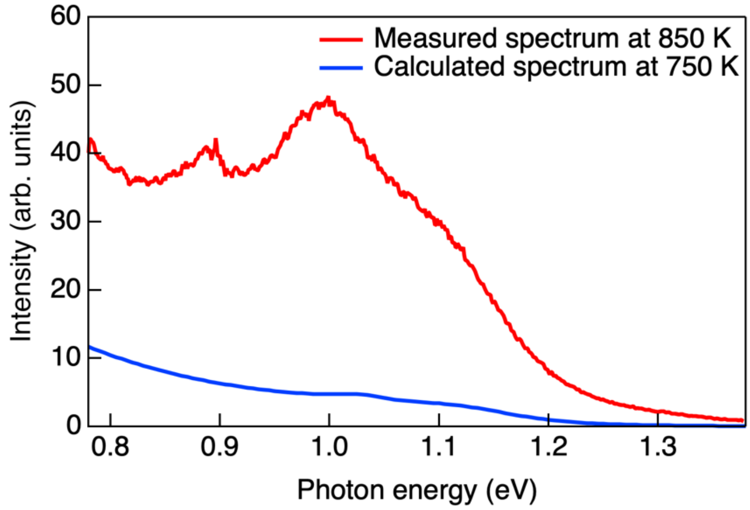

Fig. R6 | Measured thermal radiation spectrum of the free-standing CNT membrane at 850 K, together with its calculated one at 750 K.

Q3-6) Regarding the CNT film temperature estimate, I feel it would be better to cross-check the estimation, such as using a multiphysics simulation to simulate temperature profiles. The semiconducting CNT film is neither crystalline nor very conductive. The thermal conductance could be poor for the CNT film. The temperature may not be uniform inside a film.

A3-6) We thank the reviewer for the helpful comment. We simulated the temperature distribution of a free-standing SWCNT membrane heated by the tungsten heater using the Heat Transfer module of COMSOL Multiphysics software. Initially, we calculated the temperature distribution on the tungsten heater alone during current heating using a three-dimensional model. Figure R7a shows the simulation result. Here the input current value was adjusted so that the edge of the central hexagonal part reached 850 K similar with the observations. The six sides of the central hexagon have the highest temperature of approximately 850 K and maintained a nearly uniform temperature. Thus, when a free-standing SWCNT membrane is put on the heater, it is expected that the membrane has a uniform temperature at their edges. We subsequently calculated the temperature distribution for the hexagonal SWCNT membrane, setting the boundary condition of its six sides to 850 K (Fig. R7b-f). The calculation was performed using a two-dimensional model. For the thermal conductivity of the membrane, we used a typical value of an SWCNT membrane (30.4 W mK^{-1}) reported in the literature [D. Kim *et al.*, *Langmuir*, **27**, 14532 (2011)]. As shown in Fig. R7b–f, the temperature within the SWCNT membrane is predicted to reach 850 K with nearly uniform temperature distribution after 10 seconds. Finally (t = 10 s), the difference of the temperature in the membrane is only within± 0.02 K).

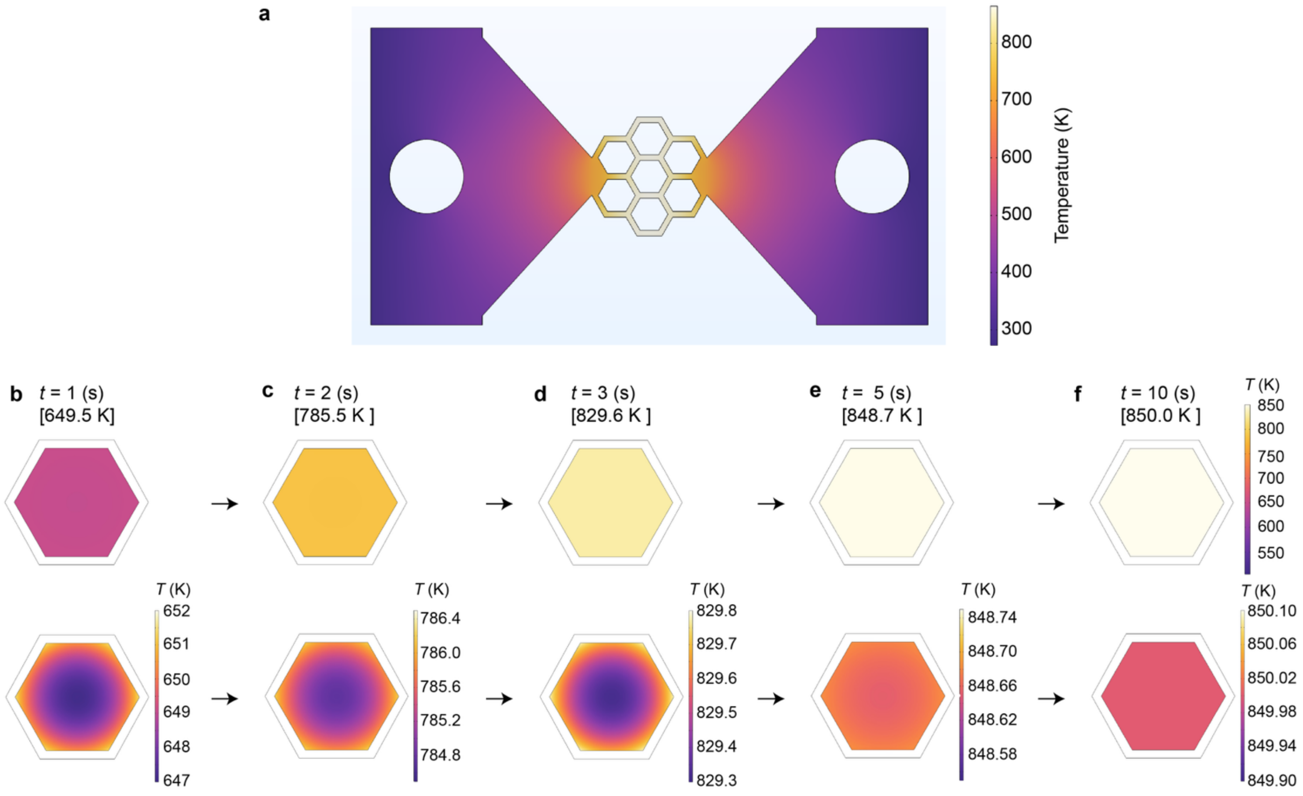

Fig. R7 | Simulated temperature distribution in a free-standing carbon nanotube membrane. **a** Temperature distribution of the designed tungsten heater. **b-f** Time-dependent temperature distribution of the carbon nanotube membrane. The upper panels are shown with a common color bar for comparison, while the lower panels use individual color bars to highlight the internal temperature distribution within each membrane at each time point.

Accordingly, we added the results shown in Fig. R7 as Supplementary Fig. 4, and a paragraph to Methods as follows:

Simulation of temperature distribution: The temperature distribution of a free-standing SWCNT membrane heated by the tungsten heater (Fig. 4a) was simulated using the Heat Transfer module of COMSOL Multiphysics. Initially, we calculated the temperature distribution of the tungsten heater alone during current heating using a three-dimensional model of the heater. Supplementary Fig. 4a shows the simulation result. Here, the input current value was adjusted so that the edges of the central hexagonal part, which corresponds to the measurement area, reached 850 K. The each side of the central hexagon has the nearly uniform temperature of approximately 850 K. Thus, when a free-standing SWCNT membrane is put on the heater, the membrane is expected to have a uniform temperature at its edges. Subsequently, we calculated the temperature distribution for the hexagonal SWCNT membrane, setting the boundary condition of its six sides to 850 K. The calculation for the membrane was performed using a two-dimensional model. For the thermal conductivity of the membrane, we used a typical value (30.4 W mK^{-1}) of SWCNT membranes reported in the previous study[53]. The emissivity of the SWCNT membrane at 850 K is deduced from the experimental results shown in Fig. 5a. Supplementary Fig. 4b–f show the results after 1–10 seconds, indicating that the temperature of the SWCNT membrane within the central hexagonal part of the heater reaches 850 K after 10 seconds with a nearly uniform temperature distribution.

Q3-7) Finally, I am curious about the selective thermal emitter based on CNTs, which the authors have argued as a future application. If we look at Fig. 5, the CNT emission is also weaker than the blackbody radiation. Would it be better to construct a selective thermal emitter by putting a narrow-band near-infrared bandpass filter after a blackbody radiator, instead of using CNTs?

A3-7) This is the fundamental, and frequently-asked question regarding the necessity of wavelength-selective thermal emitter for TPV applications. To understand this, the following two points must be noted: (1) The importance of wavelength-selective emitters varies depending on the temperature of the

high-temperature heat source. (2) There is no single interference optical filter with ideal characteristics across the entire wavelength range from far-infrared to the visible spectrum.

With regard to (1), if the temperature of the heat source is significantly high (e.g. >2500 K), the Planck distribution peak (black body radiation peak) shifts to higher energy region (~0.6 eV). Under such conditions, the fraction of the radiation power above the PV cell's band gap (e.g. 0.67 eV for GaSb) exceeds ~60%. In this regime, using an interference optical filter may also be somewhat effective, as it can reflect a significant portion of sub-bandgap photons back to the black body radiator to maintain the source temperature (photon recycling), which avoid entropy generation in the PV cell and enhance the net TPV efficiency.

However, our primary target for TPV applications is around 1200 K, where a wider variety of heat sources can be utilized. As mentioned in our response to Reviewer 2, the maximum temperature of 850 K achieved in this experiment was limited by the thermal stability of the (7,5) SWCNT with a relatively small diameter. However, 850 K is still too low for TPV applications, and we expect that applications around 1200 K will be possible using larger diameter SWCNTs. We believe that not explicitly stating this was one of the problems with the previous manuscript that casts doubt on the significance of the findings of this research. Therefore, in the revised manuscript, we have added discussion and new Figure about the pathway toward this goal and new data in Page 11, 2nd paragraph, Figs. 6 and 7).

At this moderate temperature, the use of wavelength selective emitter is generally considered essential. This is because the majority of emitted photons from the black body (about 90 % of the radiation) have energies below the PV cell's band gap. To block these photons using a filter, an interference filter capable of functioning across an ultra-wideband spectrum from ~1.8 μm to >10 μm is necessary. Within this broad range, the optical filter must achieve near-perfect reflection because any optical absorption within the filter leads to entropy generation (parasitic heating), which significantly degrades the net TPV conversion efficiency.

As mentioned in the point (2) above, practically, constructing an ideal optical filter that reflects all out-of-band photons (from 1.8 μm to >10 μm) is extremely challenging. As shown in Fig. R8, even high-quality commercial filters exhibit significant absorption in the mid-infrared due to material losses. This absorption leads to unwanted heating of the filter itself, rather than reflecting the energy back to the source.

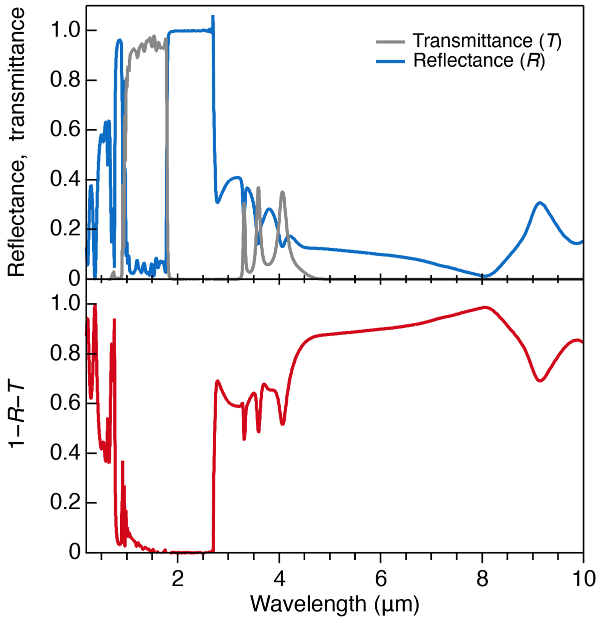

Fig. R8 | Optical spectra (Reflectance R and transmittance T) of a typical commercial optical filter (SP-1800 nm, IR System Co. Ltd), for which the specified transmission band of 1.3–1.8 µm and a blocking band of 1.8–3.0 µm. $1 - R - T$ represents the optical absorption (loss) within the filter. Clearly, outside the specified range (photonic band gap), significant absorption occurs due to the intrinsic losses of the filter materials.

In contrast, excellent selective emitters inherently suppress emission in the long-wavelength regime directly at the source. This approach eliminates the need for complex auxiliary optical components, and avoids the associated parasitic absorption losses, maximize the efficiency of TPV systems operating at moderate temperatures (~1200 K).

Clearly, in wavelength regions outside the specified range, extremely high optical absorption occurs due to losses in the filter's constituent materials. Even if this filter is placed after a blackbody radiator, most radiated photons are absorbed by the filter, causing entropy generation within it.

Instead, if there is an excellent wavelength-selective emitter that significantly reduces the low energy photon fraction in its thermal radiation, this approach avoids the losses associated with the parasitic absorption of low energy photons by various components, as well as transmission loss through the PV cell, thereby contributing to improved energy conversion efficiency via TPV operated at moderate temperatures.

In the revised manuscript, we have added a discussion stating that the spectral efficiency, defined as the ratio of the energy flux above the PV cell's bandgap to the total energy flux, can be increased up

to 0.64 even at 1200 K. This is achieved by employing (10,3) SWCNTs, which have a slightly larger diameter and higher thermal stability than (7,5) SWCNTs, in combination with the simple planar layered structure we recently proposed [Hizukuri *et al.*, *ACS Photonics* **12**, 6984 (2025)]. Once such high spectral efficiency is realized at the emitter level, the strategic integration of optical filters could further enhance the overall system performance by reflecting the remaining out-of-band photons.

Responses to the Reviewers' comments and suggestions

For Reviewer #2:

The authors largely addressed my concerns and I am able to recommend publication.

We thank the Reviewer for his/her positive evaluation of our work and for recommending it for publication in Nature Communications.

For Reviewer #3:

I thank the authors for their detailed response. I still have a fundamental question:

*(1) Regarding "thermally excited excitons" in Fig. 1b, following the authors' calculations, only a very tiny amount of thermally populated excitons is generated. Specifically, if the exciton level is ~1eV and T = 1000K, the exciton occupancy ratio exp(-E/kb*T) is ~ 0.001%. Then the thermal radiation from these excited excitons is intrinsically weak. I don't quite get how this exciton thermal emission in narrow-diameter CNTs can be useful for TPV applications around 1000 K.*

If T = 1200K, the ratio becomes 0.006%, which is still quite small. The blackbody radiation peak is ~2.42 μm, corresponding to ~0.51 eV. However, getting larger-diameter CNTs with such a small bandgap seems still quite challenging, particularly for scalable film manufacturing.

We thank the reviewer for this insightful question regarding the practical utility of exciton thermal radiation. Below, we address the concern by clarifying the difference between microscopic exciton population and macroscopic emissive power.

In brief, the thermal population of excitons at 1200 K for an exciton level of ~ 0.8 eV reaches approximately one exciton per ~ 2 μm-long CNT (Please see **Microscopic exciton population** below). This exciton population in an *individual* CNT at 1200 K may appear low. However, when considering the use of a large number of CNTs as a membrane, the ensemble integration effectively overcomes this individual-level constraint. (Please see **Macroscopic emissive power at 1200 K** below).

Microscopic exciton population at 1200 K

Given an exciton size of ~ 1 nm, a single exciton captures oscillator strength from ~10^2 carbon atoms. With a Boltzmann factor of ~ 4×10^{-4} at 0.8 eV and 1200 K, approximately one exciton (on ~10^2 carbon atoms) can be thermally excited in a 2 μm-long CNT (consisting of ~ 2×10^5 atoms). This thermal population of one exciton per ~ 2 μm-long CNT is consistent with our previous observations reported in [Nishihara *et al.*, Commun. Mater. **2025**, 6, 89], where the exciton thermal radiation intensity of an individual CNT at ~1200 K was comparable to saturated photoluminescence intensity due to strong exciton–exciton annihilation, where about one exciton per ~ 2 μm (excitation spot size) is considered to exist on average.

Macroscopic emissive power at 1200 K

The exciton population in an *individual* CNT at 1200 K may appear low. However, the utility in TPV applications in terms of emissive power arises from macroscopic factors. A TPV emitter utilizes an enormous number of CNTs. The collective emission from this ensemble, combined with the optical interference effects in the proposed few-layer structure described in our revised manuscript, significantly enhances the emissive power. As shown in Fig. 7 in the main text, the emissivity at the exciton resonance reaches ~0.8. This means that the emissive power at 0.8 eV is ~80% of the theoretical blackbody limit at that temperature, regardless of the limited occupancy per individual state.

To further demonstrate its practical utility, Figure R1 compares the thermal radiation spectrum of our proposed thermal emitter at 1200 K with the solar spectrum (AM1.5G). The integrated photon flux above a typical PV cell band gap for TPV (0.67 eV, indicated by the vertical dotted line in Fig. R1) of ~4.7×10^{22} s^{-1} m^{-2} is more than ~10 times higher than that of non-concentrated sunlight (~3.5×10^{21} s^{-1} m^{-2}), which significantly exceeds the standard threshold (Above-gap photon flux > 1-SUN photon flux) for viable TPV power generation. Additionally, since the (10,3) CNTs assumed for the emitter in Fig. R1 can be separated using current CNT structure-sorting methods [Y. Yomogida *et al.*, Nat. Commun., **2016**, 7, 12056], CNTs suitable for the proposed application are available for scalable film manufacturing.

In summary, the seemingly limited emission intensity at the individual-nanotube level is effectively compensated for by ensemble integration and structural optimization, ensuring sufficient near-infrared power density for TPV applications. Simultaneously, the excellent spectral selectivity is preserved by the intrinsic physical properties of the individual CNTs. In response to the reviewer's question, we have added Supplementary Fig. 6 in the Supplementary Information to clarify that the emissive power from the proposed structure at 1200 K is sufficiently high for practical use.

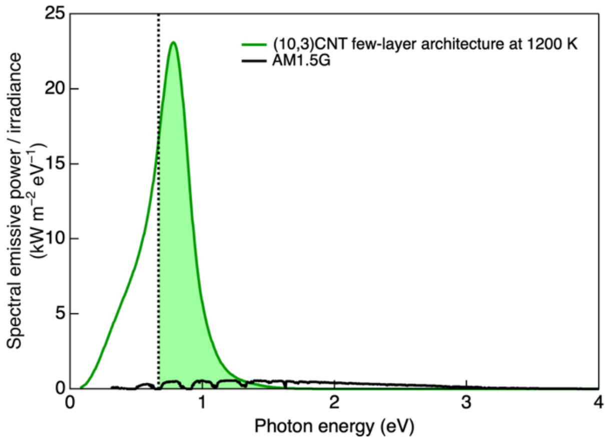

Fig. R1 | Solar spectrum (AM1.5G) and thermal radiation spectrum of (10,3) carbon nanotube (CNT) few-layer architecture at 1200 K.
