# Supplementary material for: Exciton thermal radiation from macroscale membranes composed of chirality-sorted carbon nanotubes and its control
Source: Nat Commun. 2026 Jul 29;17:6967. doi: 10.1038/s41467-026-75711-4 (PMC13421534; doi:10.1038/s41467-026-75711-4)
Supplement: Supplementary file 4 — Source Data [file 41467_2026_75711_MOESM4_ESM.zip › Source data/Description of Source Data.docx]

**Description of Additional Supplementary Files**

**File Name: Numerical_Source_Data_All_Graphs.xlsx**

Description: The numerical source data underlying all graphs presented in the main and Supplementary figures.

**File Name: Supplementary_Data_1.txt (Fig. 2c)**

Description: Absorption spectrum of the dispersion.

**File Name: Supplementary_Data_2.txt (Fig. 2d)**

Description: Reflectance spectrum of the membrane.

**File Name: Supplementary_Data_3.txt (Fig. 2e)**

Description: Reflectance spectrum of the membrane.

**File Name: Supplementary_Data_4.txt (Fig. 2f)**

Description: Transmittance spectrum of the membrane.

**File Name: Supplementary_Data_5_###K.txt (Fig. 3c)**

Description: Raw data of the transmitted signal spectrum at ### K.

**File Name: Supplementary_Data_5_###K_bg.txt (Fig. 3c)**

Description: Raw data of the transmitted background spectrum at ### K.

**File Name: Supplementary_Data_5_Ref.txt (Fig. 3c)**

Description: Reference signal spectrum.

**File Name: Supplementary_Data_5_Ref_bg.txt (Fig. 3c)**

Description: Reference background spectrum at ### K.

**File Name:** **Supplementary_Data_6_TR.txt (Fig. 4c)**

Description: Raw data of the thermal radiation spectrum.

**File Name: Supplementary_Data_6_TR_bg.txt (Fig. 4c)**

Description: Raw data of the thermal radiation background spectrum.

**File Name: Supplementary_Data_6_sensitivity.txt (Fig. 4c)**

Description: Raw data of the sensitivity curve. The corresponding background is "Supplementary_Data_6_TR_bg.txt".

**File Name: Supplementary_Data_6_sensitivity_true.txt (Fig. 4c)**

Description: Calibrated spectrum for the sensitivity curve.

**File Name: Supplementary_Data_7_heating_###K.txt (Supplementary Fig. 1a)**

Description: Raw data of the transmitted signal spectrum at ### K during the heating process.

**File Name: Supplementary_Data_7_heating_###K_bg.txt (Supplementary Fig. 1a)**

Description: Raw data of the transmitted background spectrum at ### K during the heating process.

**File Name: Supplementary_Data_7_cooling_###K.txt (Supplementary Fig. 1a)**

Description: Raw data of the transmitted signal spectrum at ### K during the cooling process.

**File Name: Supplementary_Data_7_cooling_###K_bg.txt (Supplementary Fig. 1a)**

Description: Raw data of the transmitted background spectrum at ### K during the cooling process.

**File Name: Supplementary_Data_7_Ref.txt (Supplementary Fig. 1a)**

Description: Raw data of the reference signal spectrum. The corresponding background is "Supplementary_Data_7_heating_300K_bg.txt".

**File Name: Supplementary_Data_8_heating_###K.txt (Supplementary Fig. 1b)**

Description: Raw data of the transmitted signal spectrum at ### K during the heating process.

**File Name: Supplementary_Data_8_heating_###K_bg.txt (Supplementary Fig. 1b)**

Description: Raw data of the transmitted background spectrum at ### K during the heating process.

**File Name: Supplementary_Data_8_cooling_###K.txt (Supplementary Fig. 1b)**

Description: Raw data of the transmitted signal spectrum at ### K during the cooling process.

**File Name: Supplementary_Data_8_cooling_###K_bg.txt (Supplementary Fig. 1b)**

Description: Raw data of the transmitted background spectrum at ### K during the cooling process.

**File Name: Supplementary_Data_8_Ref.txt (Supplementary Fig. 1b)**

Description: Raw data of the reference signal spectrum. The corresponding background is "Supplementary_Data_8_heating_300K_bg.txt".

**File Name: Supplementary_Data_9_heating_###K.txt (Supplementary Fig. 1c)**

Description: Raw data of the transmitted signal spectrum at ### K during the heating process.

**File Name: Supplementary_Data_9_heating_###K_bg.txt (Supplementary Fig. 1c)**

Description: Raw data of the transmitted background spectrum at ### K during the heating process.

**File Name: Supplementary_Data_9_cooling_###K.txt (Supplementary Fig. 1c)**

Description: Raw data of the transmitted signal spectrum at ### K during the cooling process.

**File Name: Supplementary_Data_9_cooling_###K_bg.txt (Supplementary Fig. 1c)**

Description: Raw data of the transmitted background spectrum at ### K during the cooling process.

**File Name: Supplementary_Data_9_Ref.txt (Supplementary Fig. 1c)**

Description: Reference signal spectrum. The corresponding background is "Supplementary_Data_9_heating_300K_bg.txt".

**File Name:** **Supplementary_Data_10.txt (Supplementary Fig. 2a)**

Description: Reflectance spectrum of the membrane.

**File Name: Supplementary_Data_11.txt (Supplementary Fig. 2b)**

Description: Reflectance spectrum of the membrane.

**File Name: Supplementary_Data_12.txt (Supplementary Fig. 2c)**

Description: Transmittance spectrum of the membrane.

**File Name: Supplementary_Data_13.txt (Supplementary Fig. 7a)**

Description: Reflectance spectrum of the few-layer structure.

**File Name: Supplementary_Data_14.txt (Supplementary Fig. 7b)**

Description: Reflectance spectrum of the few-layer structure.

**File Name: Supplementary_Data_15.txt (Supplementary Fig. 7c)**

Description: Transmittance spectrum of the few-layer structure.

**File Name: Supplementary_Data_16_heating_###K.txt (Supplementary Fig. 8a)**

Description: Raw data of the transmitted signal spectrum at ### K during the heating process.

**File Name: Supplementary_Data_16_heating_###K_bg.txt (Supplementary Fig. 8a)**

Description: Raw data of the transmitted background spectrum at ### K during the heating process.

**File Name: Supplementary_Data_16_cooling_###K.txt (Supplementary Fig. 8a)**

Description: Raw data of the transmitted signal spectrum at ### K during the cooling process.

**File Name: Supplementary_Data_16_cooling_###K_bg.txt (Supplementary Fig. 8a)**

Description: Raw data of the transmitted background spectrum at ### K during the cooling process.

**File Name: Supplementary_Data_16_Ref.txt (Supplementary Fig. 8a)**

Description: Raw data of the reference signal spectrum.

**File Name: Supplementary_Data_16_Ref_bg.txt (Supplementary Fig. 8a)**

Description: Raw data of the reference background spectrum.

**File Name: Supplementary_Data_17_heating_###K.txt (Supplementary Fig. 8b)**

Description: Raw data of the transmitted signal spectrum at ### K during the heating process.

**File Name: Supplementary_Data_17_heating_###K_bg.txt (Supplementary Fig. 8b)**

Description: Raw data of the transmitted background spectrum at ### K during the heating process.

**File Name: Supplementary_Data_17_cooling_###K.txt (Supplementary Fig. 8b)**

Description: Raw data of the transmitted signal spectrum at ### K during the cooling process.

**File Name: Supplementary_Data_17_cooling_###K_bg.txt (Supplementary Fig. 8b)**

Description: Raw data of the transmitted background spectrum at ### K during the cooling process.

**File Name: Supplementary_Data_17_Ref.txt (Supplementary Fig. 8b)**

Description: Raw data of the reference signal spectrum.

**File Name: Supplementary_Data_17_Ref_bg.txt (Supplementary Fig. 8b)**

Description: Raw data of the reference background spectrum.

**File Name:** **Supplementary_Data_18**

Description: Detailed data analysis methods for each figure.
